# Production of the antimicrobial compound tetrabromopyrrole and the *Pseudomonas* quinolone system precursor, 2-heptyl-4-quinolone, by a novel marine species *Pseudoalteromonas galatheae* sp. nov.

**DOI:** 10.1038/s41598-020-78439-3

**Published:** 2020-12-10

**Authors:** Sara Skøtt Paulsen, Thomas Isbrandt, Markus Kirkegaard, Yannick Buijs, Mikael Lenz Strube, Eva C. Sonnenschein, Thomas O. Larsen, Lone Gram

**Affiliations:** Department of Biotechnology and Biomedicine, Technical University of Denmark, Søltofts Plads 221, 2800 Kgs. Lyngby, Denmark

**Keywords:** Biologics, Environmental biotechnology, Sequencing

## Abstract

Novel antimicrobials are urgently needed due to the rapid spread of antibiotic resistant bacteria. In a genome-wide analysis of *Pseudoalteromonas* strains, one strain (S4498) was noticed due to its potent antibiotic activity. It did not produce the yellow antimicrobial pigment bromoalterochromide, which was produced by several related type strains with which it shared less than 95% average nucleotide identity. Also, it produced a sweet-smelling volatile not observed from other strains. Mining the genome of strain S4498 using the secondary metabolite prediction tool antiSMASH led to eight biosynthetic gene clusters with no homology to known compounds, and synteny analyses revealed that the yellow pigment bromoalterochromide was likely lost during evolution. Metabolome profiling of strain S4498 using HPLC-HRMS analyses revealed marked differences to the type strains. In particular, a series of quinolones known as pseudanes were identified and verified by NMR. The characteristic odor of the strain was linked to the pseudanes. The highly halogenated compound tetrabromopyrrole was detected as the major antibacterial component by bioassay-guided fractionation. Taken together, the polyphasic analysis demonstrates that strain S4498 belongs to a novel species within the genus *Pseudoalteromonas,* and we propose the name *Pseudoalteromonas galatheae* sp. nov. (type strain S4498^T^ = NCIMB 15250^T^ = LMG 31599^T^).

## Introduction

The emergence and spread of antibiotic resistant bacteria is a worldwide threat to human health^1^. Most antibiotics are of microbial origin and have been derived from terrestrial organisms^2^, however, in recent years, also marine microorganisms have been explored for novel natural products^3,4^. In 2017, 242 new natural products identified from marine bacteria were reported, and 137 of these originated from *Streptomyces* species^5^, which are well known producers of antibiotic compounds^2^. During the Danish research expedition Galathea 3, marine bacteria were isolated based on their antibacterial activity with the aim of identifying novel antimicrobial compounds^6^. Many of the isolates turned out to belong to the marine genus *Pseudoalteromonas*^6,7^*.* The genus shows a cosmopolitan occurrence in marine waters where it, on average, constitutes 2–3% of the total prokaryotic population^8^. Pseudoalteromonads are prolific biofilm formers and may repel or induce settlement of other bacteria^9^ or bivalve larvae^10^. The genus is currently sub-divided into two groups based on phenotypic and genotypic traits: non-pigmented and pigmented species. The non-pigmented species are renowned for production of hydrolytic enzymes degrading algal polymers whereas the pigmented group is known for its production of a suite of antimicrobial compounds as well as several glycosyl hydrolases^11^. Thus, the genus is important in the marine biogeochemical cycle and of interest in biotechnology. On a genomic level, pigmented species contain a large number of biosynthetic gene clusters (BGCs), often dedicating as much as 15% of their genome to production of secondary metabolites (SMs). Whilst the structure of many of these secondary metabolites have been elucidated, most are still unknown^12^.

In a recent genome-wide analysis of *Pseudoalteromonas* strains^12^, one strain (S4498^T^) stood out due to its antibacterial activity, a sweet odor and the lack of yellow pigmentation found in the closely related species (*Pseudoalteromonas piscicida*,* Pseudoalteromonas maricaloris* and *Pseudoalteromonas flavipulchra*), with which it clustered based on 16S rRNA gene sequence comparison. Strain S4498^T^ was also placed close to the species type strains based on average nucleotide identity (ANI), however, sharing less than 95% identity. ANI analysis is used to infer genetic relatedness between strains based on whole genomes, where strains are considered of the same species when more than 95% similar^13^. DNA-DNA hybridization (DDH) has for many years been the gold standard in identifying novel bacterial species, and strains showing ≥ 70% similarity are regarded as the same species^14^. The rapid advancements in genome sequencing technologies have enabled computation of genome relatedness of two or more genomes based on their complete genome sequences using tools, such as in silico DDH and ANI. Combined, these computational values have been coined as the Overall Genome Related Index (OGRI)^15^ and can be used for delineating species into known or novel species based on genome relatedness to type strains of a species. The yellow pigmented *Pseudoalteromonas* and their bioactive compounds, such as korormicins and bromoalterochromides, have been widely studied^16^. It is believed that the compound bromoalterochromide is responsible for its pigmentation^17^. Due to the marked bioactivity of strain S4498^T^, we here used a polyphasic approach combining genomic, metabolomic and phenotypic comparison to analyze the metabolomic and taxonomic relationship between S4498^T^ and related species.

## Results and discussion

### Genomic and phenotypic comparison of strain S4498^T^ and ***Pseudoalteromonas*** type strains

**Figure 1:**
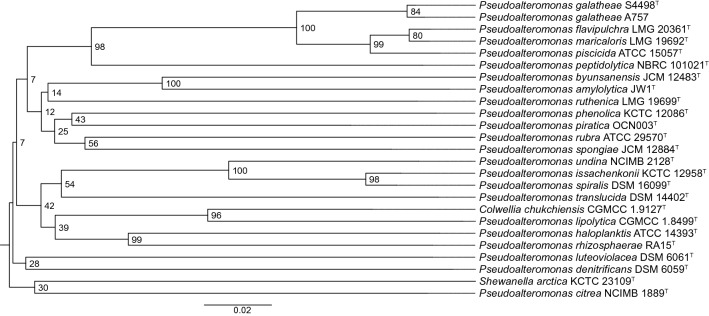
Phylogenomic tree of *Pseudoalteromonas* species based on genome distances as provided by the TYGS platform^61^. Values are pseudo-bootstraps with average branch support of 59.6%. The tree is rooted at the midpoint.

A key phenotypic difference between strain S4498^T^ and closely related type strains (Fig. 1), is the absence of yellow pigmentation (Supplementary Fig. S1). Early 2019, the genome sequence of *Pseudoalteromonas* sp. A757 was released on NCBI, and this strain yielded an ANI of 99.3% when compared with S4498^T^. Several studies have been conducted with this strain^18–21^ and it was therefore included in the analyses. Both strains were isolated in the North Atlantic; strain A757 from plastic debris^21^ and 4498^T^ from a crustacean^6^.

**Figure 2:**
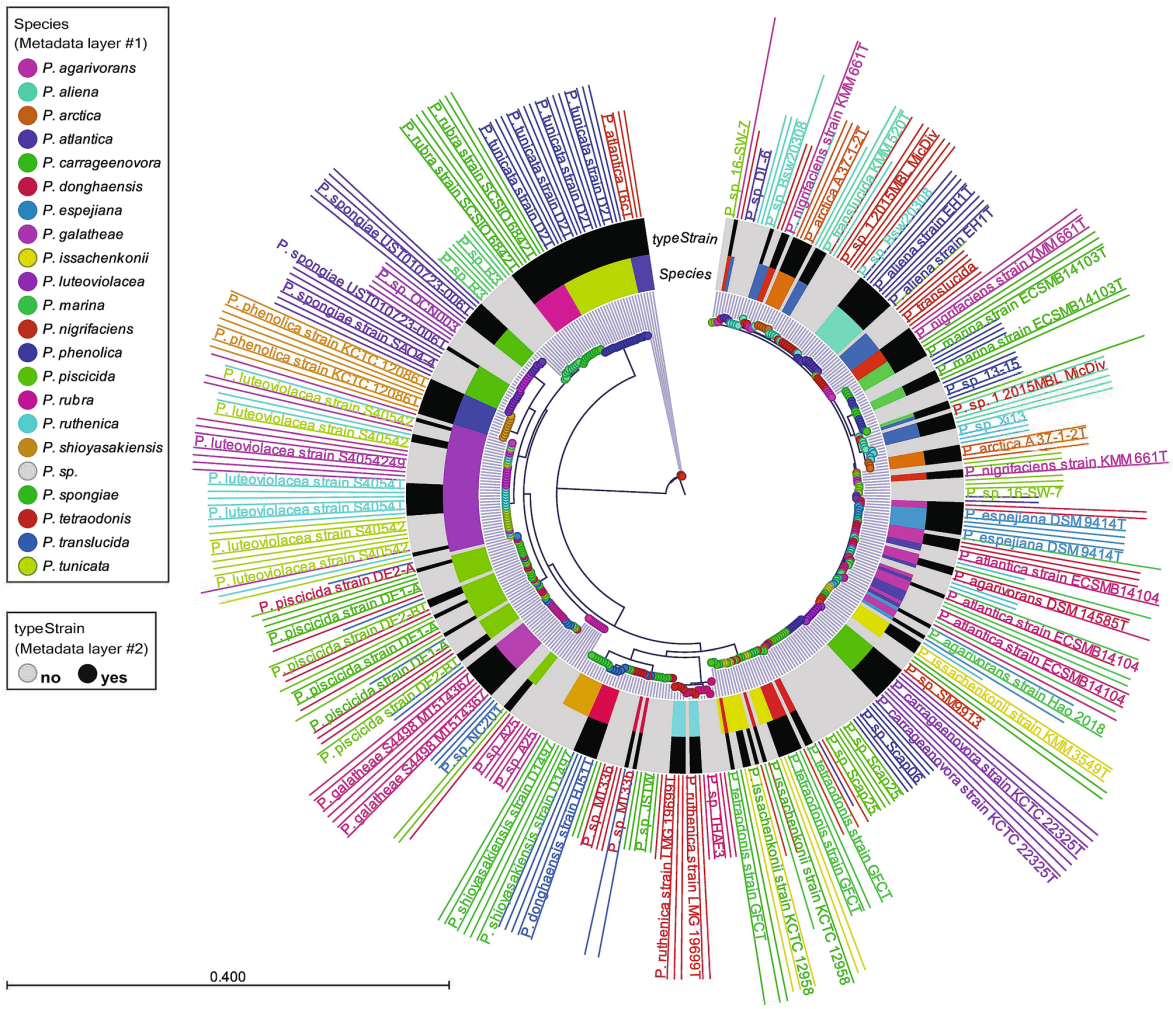
Phylogenetic tree of all alleles of 16S rRNA genes in *Pseudoalteromonas* genomes considered ‘complete’ in NCBI. Nodes and label colors correspond to individual genomes. Inner color ring and outer ring denotes species and type strain status (black = type strain, grey = no type strain), respectively. Labels have been compressed to avoid overlapping. *P. atlantica* T6c^T^ has been used as outgroup.

The type strains phylogenetically most closely related to S4498^T^ and A757, i.e. *P. piscicida* NCIMB 1142^T^ and *P. flavipulchra* LMG 20361^T^, were acquired for comparison. Additionally, to rule out that strains S4498^T^ and A757 belong to an existing species, of which the whole genomes have not been sequenced, a preliminary 16S rRNA gene sequence-based comparison of all type strains with validly published names was conducted (Supplementary Fig. S2). This identified two additional closely related, yellow pigmented species, *P. maricaloris* LMG 19692^T^ and *P. peptidolytica* DSM 14001^T^*,* that were acquired and their genomes sequenced. Genome sizes of the six strains varied from 5.1 to 5.6 Mb and G + C content varied from 42.4% to 43.3% (Table 1). Strain A757 has a genome size of 5.1 Mb and G + C content similar to that of S4498^T^ of 43%. The genome size of *P. galatheae* S4498^T^ is 5.4 Mb, organized in what is highly likely two chromosomes. The herein sequenced strains: S4498^T^, *P. peptidolytica* DSM 14001^T^, *P. flavipulchra* LMG 20361^T^, and *P. maricaloris* LMG 19692^T^ were checked for completeness and contamination using CheckM^22^ as requested in the minimal standards^23^. The genomes were of good quality; the assemblies having high completeness (99.89%, 100%, 99.89% and 99.89%, respectively) and low contamination (0.38%, 1.51%, 0.89% and 0.46%, respectively). Identification of novel species based on 16S rRNA gene sequence similarity can be flawed e.g. due to multiple alleles of the 16S rRNA gene in some species^24^. Extraction and phylogenetic profiling of all 16S rRNA genes from complete genomes of *Pseudoalteromonas* revealed a high level of variability of the gene within individual genomes as well as frequent instances of identical alleles in several genomes (Fig. 2). This variability makes the 16S rRNA gene a poor marker of species phylogeny, at least in *Pseudoalteromonas*. Previous ANI analysis indicated that S4498^T^ does not belong to any existing species in the genus, with an ANI value below 95%^12^. The ANI values between the strains from this study demonstrate that S4498^T^ and A757 belong to the same novel species as they share less than 95% ANI value with the closest related type strains (Table 2). In silico DDH estimates were well below the cut-off of 70% for all type strains (between 24.5% and 49.4% similarity) (Table 2). Hence, S4498^T^ and A757 belong to the same novel genomospecies because the ANI and dDDH values between their genomes were 99.3% and 94.3%, respectively.

**Table 1:** Genomic data on *Pseudoalteromonas* strains included in type strain comparisons.

Species	Strain	Colour	Genome size (Mb)	G + C content, %	N50	Accession no.
*P. galatheae*	S4498^T^	Light ivory	5.4	43.0	4061465	PNCO01
*P. galatheae*	A757	nd	5.1	43.0	224630	QNQN01
*P. piscicida*	NCIMB 1142^T^	Yellow	5.5	43.3	–	NZ_CP011924.1/NZ_CP011925.1
*P. flavipulchra*	LMG 20361^T^	Yellow	5.4	43.2	337863	VSSD01
*P. maricaloris*	LMG 19692^T^	Yellow	5.6	43.1	266291	WEIA01
*P. peptidolytica*	DSM 14001^T^	Pale yellow	5.1	42.4	128830	WEHZ01

*Nd* no data.

**Table 2:** In silico DNA–DNA hybridization values of four type strains of *Pseudoalteromonas* and strain A757 as compared to *Pseudoalteromonas galatheae* S4498^T^.

Species	Average nucleotide identity, %, to S4498^T^	DNA–DNA hybridization values		
		DDH estimate, %		Probability (%) that DDH > 70% (i.e. same species)
		avg	Range	
*P.* strain A757	99.3	94.2	92.3–95.5	96.99
*P. piscicida* NCIMB 1142^T^	93.0	48.6	46.0–51.3	15.57
*P. flavipulchra* LMG 20361^T^	93.2	49.4	46.8–52.0	17.41
*P. peptidolytica* DSM 14001^T^	86.0	24.5	22.2–27.0	0.01
*P. maricaloris* LMG 19692^T^	93.1	49.1	46.5–51.7	16.64

DDH values are shown using formula 2 of the DSMZ GGDC Genome-to-Genome Distance Calculator 2.1.

Assigning strains to species in the yellow pigmented *Pseudoalteromonas* group is complicated by the fact that *P. piscicida* NCIMB 1142^T^ shares 96% ANI value with *P. flavipulchra* LMG 20361^T^ and *P. maricaloris* LMG 19692^T^*,* which share 99% ANI value with each other (Supplementary Table S1). Reciprocal ANI values were identical to those in Supplementary Table S1 (data not shown), providing further robustness to the analysis. This relatedness is visualized by the in silico DDH values indicating that *P. flavipulchra* LMG 20361^T^ and *P. maricaloris* LMG 19692^T^ are the same species (DDH value 89.60%) and that *P. piscicida* NCIMB 1142^T^ is at the borderline to a separate species (DDH values to *P. flavipulchra* LMG 20361^T^ and *P. maricaloris* LMG 19692^T^ 68.70% [65.7–71.5%]) (Supplementary Table S2). Since type strains serve as key reference points in bacterial taxonomy, this should be further investigated. The “gold standard” for species description using ANI is often accepted as 95–96%^23^. The species cutoff has even been suggested at a low 94%^25^ or a high 97.1%^26^. In this case, the use of genomic information alone is not enough to determine whether *P. piscicida* NCIMB 1142^T^ should be grouped together with *P. maricaloris* LMG 19692^T^ and *P. flavipulchra* LMG 20361^T^ to form a single species.

Phenotypic characterization using the BiOLOG GEN III system grouped *P. maricaloris* LMG 19692^T^ and *P. flavipulchra* LMG 20361^T^ together, whereas *P. peptidolytica* DSM 14001^T^*, P. piscicida* NCIMB 1142^T^ and S4498^T^ all had different biochemical profiles (Supplementary Table S3). The carbohydrate active enzymes, subclass glycosyl hydrolases (GHs), were predicted using a HMMER search with dbCAN2^27^. The GH profiles, although quite similar, clustered the strains into groups (Supplementary Fig. S3). Strains S4498^T^ and A757 had identical GH profiles and grouped together with *P. peptidolytica* DSM 14001^T^. *P. piscicida* NCIMB 1142^T^*, P. maricaloris* LMG 19692^T^ and *P. flavipulchra* LMG 20361^T^ formed another group mainly due to an increased number of GH3, GH13, GH16 and GH20 enzymes. *P. peptidolytica* DSM 14001^T^ was the most unique strain in terms of GH profile in agreement with the ANI values, according to which *P. peptidolytica* DSM 14001^T^ is the least similar to the other five strains. Based on our phylogenomic and phenotypic analyses, *P. maricaloris* and *P. flavipulchra* should be combined as one species.

### Biosynthetic gene cluster analysis

In silico prediction of biosynthetic gene clusters (BGCs) was done using antiSMASH version 5.0^28^ (Table 3). In total, eight BGCs were identified in both S4498^T^ and A757 and the BGCs in these two strains were identical. The BGC encoding enzymes involved in the biosynthesis of the yellow pigment alterochromide, were only identified in the four type strains, but not in S4498^T^ and A757, and a manual BLAST search for the alterochromide cluster using the MIBiG identifier BGC0000299 was conducted in the two genomes with no positive hits. Chemical profiling (see below) allowed identification of two additional BGCs not identified by antiSMASH, encoding enzymes required for the production of tetrabromopyrrole (TBP) and pseudane III–X, including 2-heptyl-4-quinolone (HHQ; also referred to as pseudane VII). Manual BLAST searches using the query genes for TBP and HHQ production were conducted for all strains. The *bmp* gene cluster (including *bmp1–bmp4* and *bmp9–bmp10* responsible for TBP production in strain A757^29^) was identified in S4498^T^ and *P. peptidolytica* DSM 14001^T^. Genes encoding enzymes required for HHQ production (*pqsA-pqsE, phnA* and *phnB)* were only identified in S4498^T^ and A757^21^. No BGC was shared between all six strains, however, the BGC predicted by antiSMASH to encode enzymes required for biosynthesis of an *N*-tetradecanoyl tyrosine-like compound was identified in all strains, except for *P. maricaloris* LMG 19692^T^*.* Since only two of the genomes are complete genomes, it is likely that this BGC would also be identified in *P. maricaloris* LMG 19692^T^*,* as BGCs are known to be difficult to detect in contig-based genomes due to the repeat regions in BGCs^30^. The predicted BGCs in all six strains shared little to no sequence homology to already known BGCs (data not shown). The BGC encoding enzymes required for the production of a serobactin-like compound was only identified in S4498^T^ and A757.

**Table 3:** Genome analyses of *P. galatheae* strain S4498^T^, A757 and four *Pseudoalteromonas* type strains using antiSMASH 5.0 and BLAST.

Cluster	Detection of cluster (+) and % homology					
	S4498^T^	A757	*P. piscicida* NCIMB 1142^T^	*P. flavipulchra* LMG 20361^T^	*P. maricaloris* LMG 19692^T^	*P. peptidolytica* DSM 14001^T^
Tetrabromopyrrole^a^	+	+				+
Pseudane^a^	+	+				
Alterochromides			+ (95%)	+ (95%)	+ (95%)	+ (95%)
Amonabactin			+ (42%)			
Serobactin	+ (23%)	+ (23%)				
*N*-tetradecanoyl tyrosine	+ (6%)	+ (6%)	+ (6%)	+ (6%)		+ (6%)
Ikarugamycin					+ (8%)	

For antiSMASH, only BGCs of complete clusters are considered. + represents presence of the cluster.

^a^Detected using BLAST, not identified in antiSMASH.

**Figure 3:**
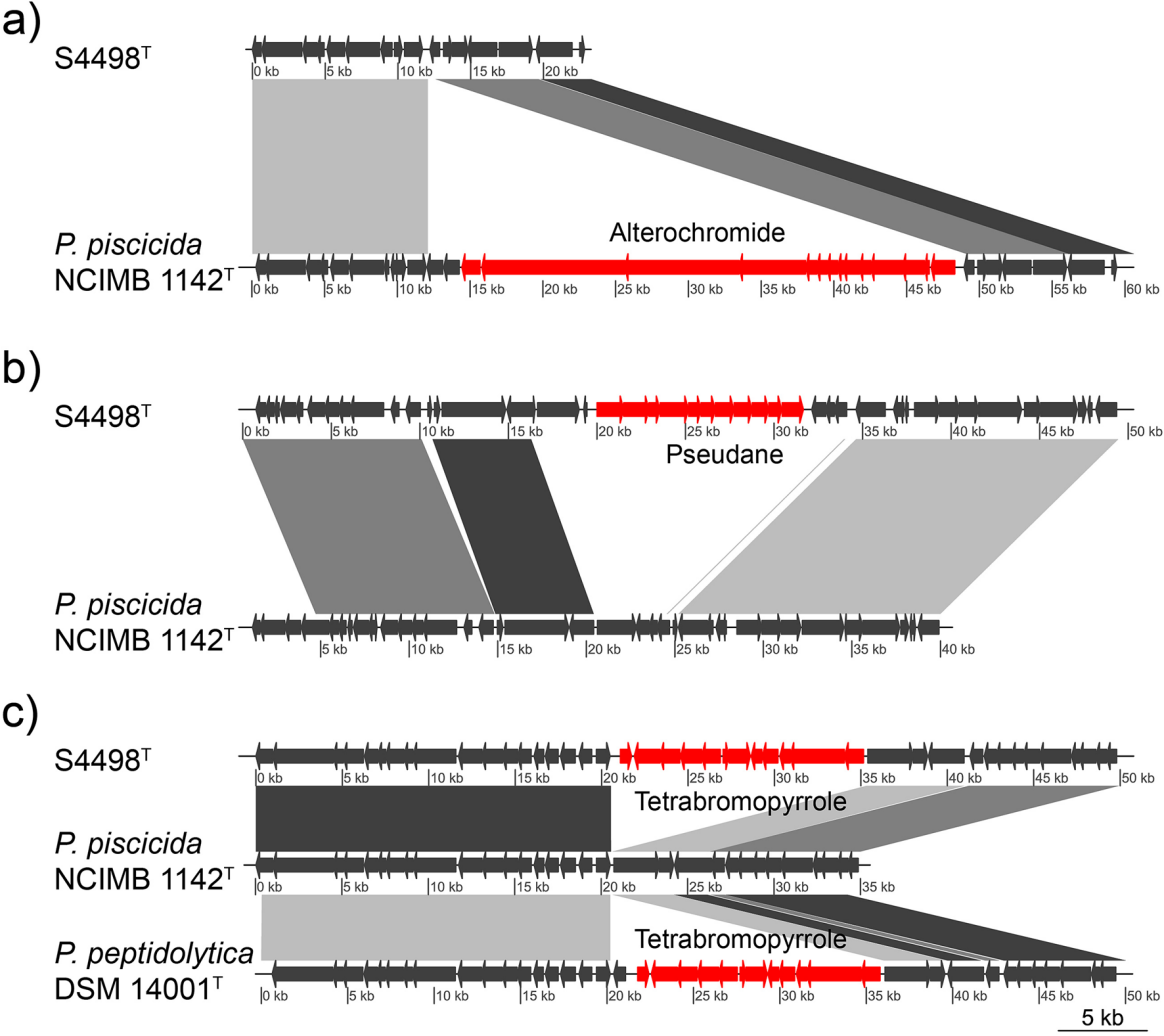
Synteny comparisons of genes surrounding (**a**) the alterochromide cluster in *P. piscicida* NCIMB 1142^T^ compared to *P. galatheae* S4498^T^, (**b**) the pseudane cluster in strain S4498^T^ compared to *P. piscicida* NCIMB 1142^T^ and (**c**) the tetrabromopyrrole cluster in *P. galatheae* S4498^T^ and *P. peptidolytica* DSM 14001^T^ as compared to *P. piscicida* NCIMB 1142^T^*.* Biosynthetic gene clusters are marked with red and differently grey-shaded bars display homology between regions.

BGCs are commonly subject to horizontal gene transfer (HGT) in bacteria^31^, and it was investigated if key biosynthetic genes had likely been subjected to HGT. Synteny of genes surrounding the alterochromide, pseudane and *bmp* cluster was compared in the genomes of S4498^T^ and *P. piscicida* NCIMB 1142^T^. The genes surrounding the alterochromide cluster in *P. piscicida* NCIMB 1142^T^ were also present in the same order in strain S4498^T^, suggesting that this cluster has been lost in an evolutionary event (Fig. 3a). The opposite was the case for the pseudane cluster, which clearly seems to have been acquired by strain S4498^T^, when compared to the surrounding genes in *P. piscicida* NCIMB 1142^T^ (Fig. 3b). The *bmp* cluster was also identified in *P. peptidolytica* DSM 14001^T^ and was therefore investigated for synteny of genes surrounding the *bmp* cluster and compared to *P. piscicida* NCIMB 1142^T^*.* Of the type strains in the yellow pigmented cluster, *P. peptidolytica* DSM 14001^T^ is most distantly related to S4498^T^, and the *bmp* cluster has the same insertion site in S4498^T^ and *P. peptidolytica* DSM 14001^T^ as compared to *P. piscicida* NCIMB 1142^T^ (Fig. 3c). This observation is similar to findings in the genus *Salinispora*, in which specific hotspots for insertion of BGCs are present in the genome^32^. BGCs have been described as being “selfish operons” for which exchange events are favored between closely related strains^33^. This explains the acquisition of the *bmp* cluster by S4498^T^ and A757, since it is shared between several species of *Pseudoalteromonas*^29^*.* Extensive BGC diversity has been seen even among closely related strains, likely as a strategy of maximizing the BGC potential and minimizing the load of a single strain^31,34^. This could be a reason for the successful speciation event of *P. galatheae* as strains of the species could benefit from the products formed by other yellow pigmented strains without the production cost itself.

### Chemical profiling of strain S4498^T^

**Figure 4:**
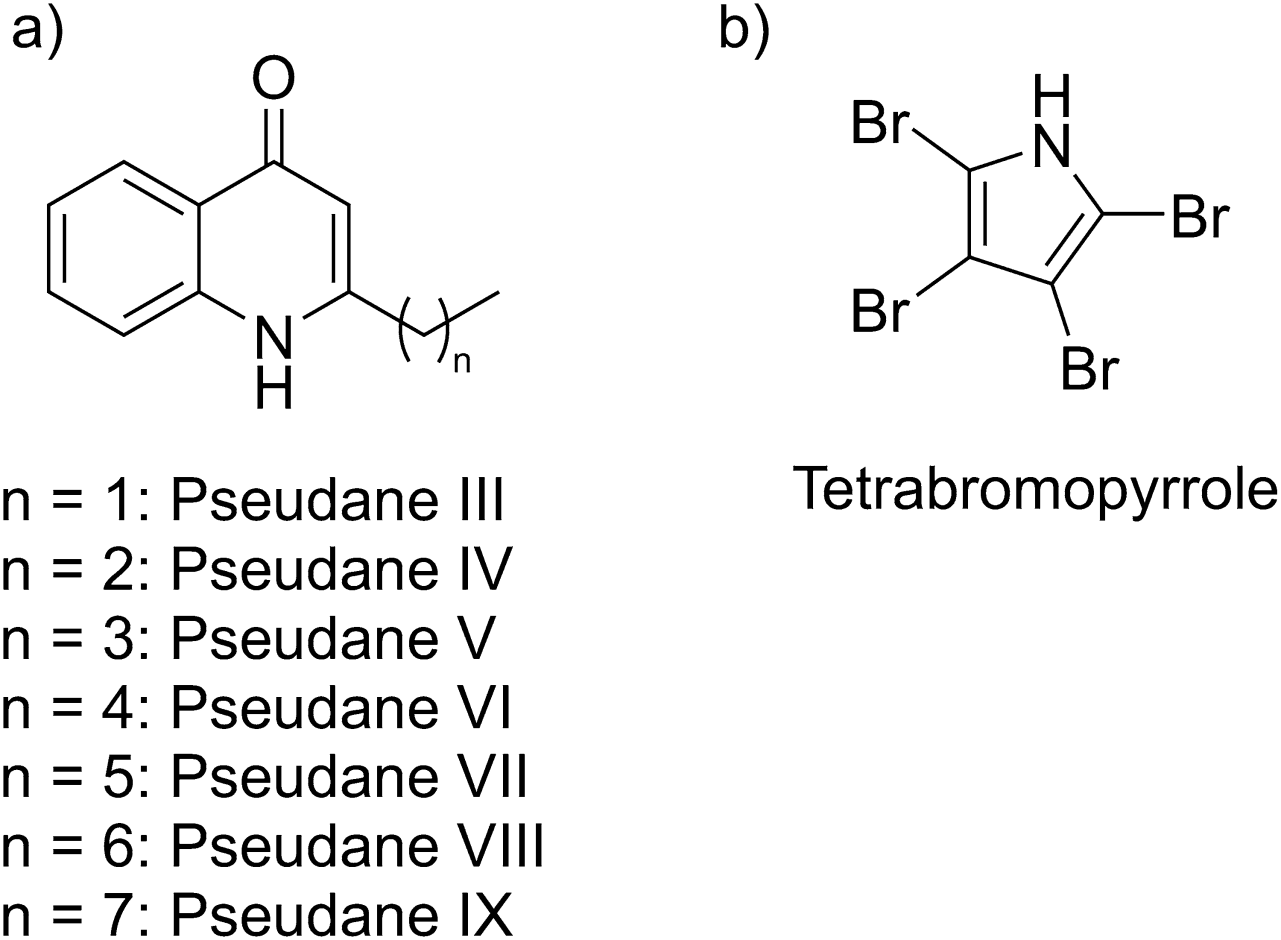
Structures of the major identified secondary metabolites detected from *P. galatheae* S4498^T^. (**a**) Pseudanes III–IX. The structures of pseudane V and pseudane VII were confirmed by NMR spectroscopy, and the identities of the remaining pseudanes were determined by similarities using their MS/MS fragmentation patterns. (**b**) Structure of tetrabromopyrrole.

Strain S4498^T^ produced a series of quinolones known as pseudanes (Fig. 4a), the halogenated TBP (Fig. 4b), and several unknown metabolites. One of the pseudanes, pseudane VII, has also been reported from strain A757^21^. In positive ionization mode, a total of seven pseudanes (III–X) could be identified from S4498^T^. The main components, pseudane V and pseudane VII were purified and their structures were confirmed by NMR spectroscopy (Supplementary Information S1). The identities of the remaining pseudanes were based on comparison of MS/MS fragmentation patterns. Additionally, some pseudane analogues were also detected. Specifically, two analogues of pseudane V and VII (*m/z* 214.1228 and *m/z* 242.1538) containing unsaturated side chains were observed, so called pseudenes^35,36^. The position of the additional double bond adjacent to the quinolone ring is in agreement with the observed shift in the absorption maximum, corresponding to the increase in the size of the chromophore and the MS/MS fragmentation pattern (Supplementary Information S2) is in agreement with previously published results^35^.

Pseudanes are commonly reported from *Pseudomonas aeruginosa* as their tautomeric 2-alkanyl-4-quinoline forms, such as HHQ. In *Pseudomonas,* HHQ acts as a precursor of the quorum sensing molecule 2-heptyl-3-hydroxy-4-quinolone, which is designated the *Pseudomonas* quinolone signal (PQS)^37^. PQS is not produced by S4498^T^, and has not been found in strain A757^21^, which is in agreement with the absence of the *pqsH* gene responsible for production of PQS in *Pseudomonas*. Pseudanes have also been detected in two other strains without available genome sequences, *Pseudoalteromonas* sp. M2^38^ and *Pseudoalteromonas* sp. B030a^21^. These strains are distantly related to each other based on 16S rRNA gene sequence similarity, as strain M2 clusters with *Pseudoalteromonas neustonica* and strain B303a clusters with *Pseudoalteromonas espejiana*, which are not related to S4498^T^ and A757 (data not shown). No antibiotic activity was found in the pseudane containing fractions from preparative HPLC of the chitin-grown culture. However, a concentration of 10 µM HHQ has antibacterial activity and can reduce biofilm formation^39^. The natural function of pseudanes in S4498^T^ and pseudane VII in A757 is not known, but in a recent mesocosm experiment, the addition of HHQ changed microbial diversity, particularly favoring an increased abundance of Alpha- and Gammaproteobacteria, including species of genus *Pseudoalteromonas*^19^. HHQ produced by A757 induces mortality in the coccolithophore *Emiliania huxleyi* and could thus mediate phytoplankton population dynamics^21^.

Strain S4498^T^ was characterized by a sweet fruity odor, which has not been reported from any other pseudane-producing *Pseudoalteromonas*. However, *P. aeruginosa* produces the well-known volatile 2-aminoacetophenone (2-AA) that is used a breath biomarker for *P. aeruginosa* in cystic fibrosis^40^. In *P. aeruginosa*, 2-AA is produced from the same pathway as HHQ and PQS, and 2-AA was also identified in the reversed phase flash chromatography fraction eluting just before pseudane V from S4498^T^. The identity of 2-AA was confirmed by comparison by LC–MS to an authentic standard. 2-AA produced by *P. aeruginosa* has been reported to activate the quorum-sensing response regulator LuxR in *Vibrio fischeri*, and it is suggested that 2-AA acts as an inter-species signal, as 2-AA does not activate the quorum-sensing systems in *P. aeruginosa* itself^41^.

TBP was responsible for the antibacterial activity as determined in bioassays. TBP is an intermediate compound from the *bmp* pathway with pentabromopseudilin as the end product^29^. Pentabromopseudilin was not detected in S4498^T^ (or strain A757), which is explained by the lack of genes *bmp5-8*. TBP induces cellular stress and mortality in some phytoplankton species^20^, and can act as a cue for coral larval settlement^42^. Hence, TBP does not only act as an antimicrobial compound, but also as a signaling molecule for some aspects of marine life. The bioactive fraction as separated by reversed phase flash chromatography also contained small amounts of additional halogenated pyrroles, namely, tribromochloropyrrole and dichlorodibromopyrrole, the tribrominated tribromopyrrole and dibromomaleimide (Supplementary Information S3). The halogenated pyrroles were detected in negative ionization mode exclusively, and while only a single isomer was detected, these compounds were detected in trace amounts, and the exact positions of the bromine and chlorine atoms have not been elucidated. The halogenase, Bmp2, responsible for the bromination has been characterized in detail^43^, and the detection of chlorinated versions in strain S4498^T^, suggests that *bmp2* is not specific to bromination but is also able to incorporate chlorine, although to a much lower extent. Fractions containing dibromomaleimide, also referred to as 3,4-dibromopyrrole-2,5-dione, had antibacterial activity. Dibromomaleimide has been identified in strain A757 as a potent efflux pump inhibitor that enhances the effect of other antimicrobials^18^. We suspect dibromomaleimide to be an oxidized by-product of TBP, as TBP was found in much higher quantities in strain S4498^T^ and is comparable to data from strain A757^18^.

Strain S4498^T^ has antibacterial activity and both genomic and phenotypic analyses revealed a potent chitinolytic machinery with four chitinases^12^. Carbon source, e.g. chitin, can influence secondary metabolite production in some bacteria^44–46^, and the production of TBP and pseudanes was investigated on five different carbon sources: mannose, chitin, glucose, the chitin monomer *N*-acetylglucosamine (NAG) and marine broth (MB), and Dunnett’s test was used for significance testing. Pseudane production (using pseudane V as proxy) per cell unit was similar on mannose as compared to chitin (P = 0.14495), whereas the production of pseudane V was significantly lower on MB, glucose and NAG, with a production of ~ 50% (P = 0.00211), 5% (P = 0.00007) and 37% (P = 0.00050) compared to mannose, respectively (Supplementary Fig. S4). TBP was produced in high concentration on MB being close to double (~ 160%) of the level produced on mannose (P = 0.03711). TBP production was reduced on chitin and glucose with a production of ~ 31% (P = 0.01793) and 9% (P = 0.00330) compared to mannose, respectively. Thus, we do not see an increase in production of antimicrobial compounds when S4498^T^ was grown in chitin medium, as was the case for strains of the Vibrionaceae family^45^. The decrease of TBP in chitin medium could indicate that the role of TBP in chitin-containing environments is not of antimicrobial character.

### Chemical profiling of strain S4498^T^ and ***Pseudoalteromonas*** type strains

HPLC–DAD–HRMS data from cultivations of S4498^T^ and four *Pseudoalteromonas* type strains grown on mannose, chitin and MB were compared by both manual inspection and using an in-house database of known SMs from the genus *Pseudoalteromonas* obtained from Antibase (Wiley) and Reaxys (Elsevier). Bruker TargetAnalysis 1.3 (Bruker Daltonics) was used to search against the in-house database. All type strains produced alterochromides and their brominated and dibrominated analogs, while *P. peptidolytica* also produced korormicin as well as TBP (Table 4, Supplementary Information S4, S5). Strain S4498^T^ did not produce any alterochromides nor korormicin under any growth condition. The alterochromide compounds are responsible for the pigmentation in the yellow *Pseudoalteromonas*^17^, supporting the observed absence of pigmentation in S4498^T^. Pseudanes were only detected in S4498^T^.

**Table 4:** Known secondary metabolites detected in *P. galatheae* S4498^T^ and four *Pseudoalteromonas* type strains based on UHPLC-DAD-HRMS/MS analysis, as well as reported metabolites from strain A757.

Compound	Presence of compound ( +) in *Pseudoalteromonas* species					
	*P. piscicida* NCIMB 1142^T^	*P. flavipulchra* LMG 20361^T^	*P. peptidolytica* DSM 14001^T^	*P. maricaloris* LMG 19692^T^	S4498^T^	A757
Alterochromide A/A′	+	(+)	(+)	(+)		
Bromoalterochromide A/A′	+	+	+	+		
Dibromoalterochromide A/A′	(+)	(+)	(+)	(+)		
Alterochromide A″	+					
Bromoalterochromide A″	+	+	+	+		
Alterochromide B/B′	+	(+)				
Bromoalterochromide B/B′	+	+	(+)	(+)		
Dibromoalterochromide B/B′	(+)	(+)	(+)	(+)		
Alterochromide B″	(+)					
Korormicin			+			
Pseudanes (III–IX)					+	+^a^
Bromopyrroles			+		+	+^b^
Dibromomalemide					+	+

+ = detected; (+) = detected in trace amounts.

^a^Only pseudane VII has been reported^21^.

^b^Only tetrabromopyrrole (TBP) has been reported^20^.

## Concluding remarks

Many strains of the genus *Pseudoalteromonas* that produce bioactive compounds have not been assigned to any existing species. Correct classification is important for understanding ecology and biodiversity and eases scientific communication. We and others have previously reported discrepancies in species delineation in the genus^12,29^ and this study also provides an example of species discrepancy between the species *P. maricaloris* and *P. flavipulchra.* This study shows that traditional species descriptions using phenotypic information can be misleading for the genus *Pseudoalteromonas* since our analyses suggest that *P. maricaloris* and *P. flavipulchra* should be combined as one species. Here, we have described a novel species belonging to genus *Pseudoalteromonas* using the minimal standards for the use of genome data for the taxonomy of prokaryotes^23^ and combined it with phenotypic and chemical profiling of the species and the closest related type strains. Strain S4498^T^ is a representative of a new species, *Pseudoalteromonas galatheae*, to which A757 also belongs and the release of the *Pseudoalteromonas* sp. A757 genome and extensive chemical profiling^18–21^ have enabled us to describe a novel species based on a polyphasic approach including genomic, phylogenetic, phenotypic and chemo-taxonomic analyses.

### Description of *Pseudoalteromonas galatheae* sp. nov.

*Pseudoalteromonas galatheae* (ga.la.the’ae. N.L. gen. n. *galatheae* referring to the research expedition on which the type strain was isolated). Cells are Gram-negative, motile rods of approx. 3 × 1 µm, and colonies on Marine Agar are 2–3 mm round and white. No yellow pigment is produced. Growth occurs between pH 6.0 and 9.5 and in a range of 10 to 42 °C. Salinity is tolerated between 0.5% and 10% (w/v). The species is catalase and oxidase positive. The species uses a wide range of carbon-sources including monomeric sugars, amino acids and polymers such as gelatin, pectin and Tween80. The species harbors several glycosyl hydrolases, including GH18 and 19 (chitinases) but does not degrade agar or carrageenan (GH16). The genome has a size of 5.4 Mb, a G + C content of 43% and is available from NCBI under accession number PNCO02.

The type strain is *P. galatheae* S4498^T^ (NCIMB15250^T^ = LMG31599^T)^ and was isolated from a crustacean in the North Atlantic Ocean.

## Materials and methods

### Bacterial strains, media and growth conditions

*Pseudoalteromonas galatheae* S4498^T^ was isolated on the Galathea 3 expedition from a crustacean in the North Atlantic Ocean (24.9963, − 67.0263)^6^. Chemotaxonomic and phenotypic comparisons included the type strains *P. piscicida* NCIMB 1142^T^ (obtained from the National Collection of Industrial Food and Marine Bacteria in United Kingdom), *P. flavipulchra* LMG 20361^T^ (obtained from the Belgian Co-ordinated Collections of Microorganisms), *P. maricaloris* LMG 19692^T^ (obtained from the Belgian Co-ordinated Collections of Microorganisms) and *P. peptidolytica* DSM 14001^T^ (obtained from the DSMZ German Collection of Microorganisms and Cell Cultures GmbH). Chemical data from *Pseudoalteromonas* strain A757^18–21^ were included. Strains were grown at 25 °C at 200 rpm to a cell density of 10^9^ CFU/ml in sterile filtered marine broth (MB; Difco 2216) or in marine minimal medium^47^ (MMM) with 0.2% w/v mannose or 0.2% w/v crystalline chitin (Sigma-Aldrich, C7170) and 1.5 w/v % casamino acids. In biological triplicates, single colonies of each strain were used to inoculate 5 ml overnight (ON) culture, after which 5 µl ON culture was used to prepare new 5 ml overnight cultures. After 24 h the ON cultures were used to inoculate 50 ml medium in 250 ml Erlenmeyer flask at a starting cell density of approximately 10^3^ CFU/ml. Samples for HPLC–DAD–HRMS analysis and antibacterial assays (described below) were taken when the cultures reached a cell density of 10^9^ CFU/ml. For fractionation and purification of compounds from S4498^T^, the cultivation on chitin was done in 8 × 1 l volumes. Phenotypic comparisons of the strains were performed on solid medium by streaking the overnight cultures on marine agar (Difco 2216), grown at 25 °C for two days. For extended phenotypic characterization of the novel species, the temperature growth range was tested on MA at temperatures from 5 °C to 44 °C. Growth at different salinities was tested in media consisting of 0.3% w/v casamino acid and 2% w/v mannose supplemented with Sea Salts (Sigma, S9883) in the range of 0–10% w/v. The ability to grow at different pH values was tested by adjusting the pH of MB medium from 4 to 11. Gram-reaction and catalase were assayed with the 3% KOH^48^ and 3% H_2_O_2_^49^ methods, respectively. Oxidase activity was tested using BBL DrySlide Oxidase kit (BD, 231746) following the manufacturer’s instructions.

### BiOLOG characterization

Phenotypic characterization was performed for all strains using the BiOLOG GEN III plates (catalog no. 1030). The protocol was modified to fit marine bacteria as follows: Strains were streaked from freeze stock on MA, and one single colony was inoculated in 10 ml inoculation fluid IF-A (catalog no. 72401) supplemented with 1 ml 20% Sea Salt solution. 100 µl suspension was pipetted into each well and the plate incubated at 25 °C for 24 h, after which the plates were read in a plate reader and analyzed according to the manufacturer’s protocol.

### Isolation of gDNA and genome sequencing

Strain S4498^T^, *P. flavipulchra* LMG 20361^T^*, P. peptidolytica* DSM 14001^T^ and *P. maricaloris* LMG 19692^T^ were grown overnight in sterile filtered MB. The genome sequence of the type strain of *P. flavipulchra* LMG 20361^T^ is deposited in the NCBI database, but has been excluded from RefSeq and marked “untrustworthy as type” and the type strain was therefore sequenced in this study. High purity genomic DNA was isolated using the QIAGEN Genomic-tip 20/G (QIAGEN, 10233) and Genomic DNA Buffer set (QIAGEN, 19060). Quantification was done on DeNovix DS-11 + 112 Spectrometer (DeNovix, USA) and Qubit 2.0 analyser with the Qubit dsDNA HS Assay Kit (Invitrogen, United Kingdom). *P. flavipulchra* LMG 20361^T^*, P. maricaloris* LMG 19692^T^ and *P. peptidolytica* DSM 14001^T^ were sequenced at the Novo Nordisk Center for Biosustainability (Technical University of Denmark, Lyngby, Denmark) using 300 bp paired-end sequencing on an Illumina MiSeq sequencer using the MiSeq V3 2 × 300 sequencing kit. Genomes were assembled using the Shovill pipeline (https://github.com/tseemann/shovill) which uses SPAdes at its core^50^ and resulted in contig-based draft genomes (68, 88 and 98 contigs, respectively). The genome of S4498^T^ was also sequenced using the MinION sequencer (Oxford Nanopore Technologies) using the EXP-FLP001flow cell priming kit, SQK-RAD004 sequencing kit, FLO-MIN106 R9 flow cell and the associated protocol (version RSE_9046_v1_revB-17Nov2017). The near closed genome was obtained using raw Illumina reads obtained from a previous study^12^ and the MinION reads. The MinION reads were filtered using the Filtlong pipeline (https://github.com/rrwick/Filtlong) and the genome was assembled using the Unicycler pipeline^51^ (https://github.com/rrwick/Unicycler) and annotated using prokka^52^. Quality assessment of the genomes was performed using QUAST^53^ (https://github.com/ablab/quast). Completeness and contamination degree were calculated using CheckM^22^. The final assembly consisted of a smaller and circularised chromosome (1,247,572 bp) as well as a larger chromosome scaffold consisting of a 4,061,465 bp contig, a 51,009 bp contig and seven contigs of less than 2500 bp. Genomes are available at the National Center for Biotechnology Information (NCBI) under the accession numbers PNCO02, VSSD01, WEIA01 and WEHZ01. *Pseudoalteromonas galatheae* S4498^T^ is available upon request and the genome available in public databases. The genome of *Pseudoalteromonas* strain A757^29^ was downloaded from NCBI (WGS accession QNQN01).

### In silico type strain comparison

Suspecting that S4498^T^ was a novel species, we compared the genome to all *Pseudoalteromonas* genomes in NCBI^12^. Using an ANI approach, strain A757 was highly similar to 4498^T^ with 99.3% identity. Based on previous ANI^12^, S4498^T^ clustered within the group of *P. piscicida*, *P. elyakovii* and *P. flavipulchra*. The type strain of *P. elyakovii* is described as a non-pigmented species^54^ and has been excluded from RefSeq and marked ‘untrustworthy as type’ in the NCBI database. Likewise, *P. flavipulchra* is also marked as ‘untrustworthy as type’. Since *P. elyakovii* is a non-pigmented species and by 16S rRNA gene sequence similarity (see below) was correctly clustered in the non-pigmented group, it was not included in our analysis. The type strain of *P. flavipulchra* LMG 20361^T^ was acquired and sequenced (described above). Only 37 of 47 species with standing in prokaryotic nomenclature (List of Prokaryotic Names with Standing in Prokaryotic Nomenclature [LPSN]^55^) are represented by their genome sequence in NCBI by start 2019, and *P. galatheae* S4498^T^ could potentially belong to hitherto uncharacterized species. A phylogenetic tree with 1000 bootstrap replications was constructed in CLC Main Workbench ver. 7.0.3 using the Neighbor joining tree construction method and Jukes-cantor nucleotide distance method, consisting of all 16S rRNA gene sequences of S4498^T^, A757 and the 47 type strains of species with validly published names. The analysis showed that two species (*P. peptidolytica* and *P. maricaloris*) that have not been genome sequenced clustered in the yellow pigmented group. Also, by 16S rRNA gene sequence analyses *P. elyakovii* was correctly clustered in the non-pigmented group. Hence, the type strains of *P. peptidolytica* DSM 14001^T^ and *P. maricaloris* LMG 19692^T^ were acquired and genome sequenced (see above). Full-length 16S rRNA gene sequences were extracted from the genome sequences of the six strains and sequence similarity was compared. ANI was determined for the six genomes (include strain A757) with the pyani pipeline^56^ using the MUMmer method. Reciprocal ANI values were calculated using OrthoANI^57^. DDH values were calculated using formula 2 of the genome-to-genome distance calculator 2.1^58^. Prediction of glycosyl hydrolases was performed with the web-based tool dbCAN2 using the hidden markov model database^27^ and clustering was done with the pheatmap package in R^59,60^ using the ‘complete’ clustering algorithm^59^.

### Phylogenomic analysis

The genomes of strain S4498^T^, A757 and *P. maricaloris* LMG 19692^T^ were uploaded to the Type (Strain) Genome Server (TYGS) platform (https://tygs.dsmz.de)^61^ and the tree based on genomic distances generated.

### Comparison on 16S rRNA alleles

All *Pseudoalteromonas* genomes marked as ‘complete’ in NCBI were downloaded using ncbi-genome-download (https://github.com/kblin/ncbi-genome-download), and individual alleles of the 16S rRNA gene were extracted from each using barrnap (https://github.com/tseemann/ barrnap). The resulting genes were then aligned with MUSCLE^62^ and a phylogenetic tree was generated from this alignment with FastTree2^63^ using the –gtr argument.

### Analysis of biosynthetic gene clusters in *Pseudoalteromonas* genomes

The six strains were submitted for BGC analysis in antiSMASH version 5.0^28^. The *bmp* gene cluster was identified using the BLAST function in CLC using sequences from *P. phenolica* O-BC30 (accession KF540211.1) as query and 2-heptyl-4-quinolone (HHQ) was identified using the gene cluster from A757 (accession KT879191-KT879199). The genomes of S4498^T^ and A757 were investigated for presence of the alterochromide gene cluster using the genes from MIBiG database^64^ with accession number BGC0000299. Genbank files from AntiSMASH analyses were used to calculate the proportion of the genomes occupied by BGCs. A BLASTp search of the proteins surrounding the alterochromide cluster (*P. piscicida* NCIMB 1142^T^)*,* pseudane cluster (S4498^T^) and *bmp* cluster (S4498^T^ and *P. peptidolytica* DSM 14001^T^) was conducted to identify regions of homology in genomes not carrying the respective clusters. Regions with similar gene arrangements were identified in all three strains, and nucleotide regions of approximately 50 kb were selected for pairwise nucleotide alignments in the NCBI webbrowser-based discontiguous megablast program. Alignments were visualized using the GenoplotR package in R^60,65^.

### Extraction of secondary metabolites

Extracts of S4498^T^ and the four type strains grown in either 0.2% (w/v) mannose, 0.2% (w/v) chitin or sterile filtered MB were produced in biological triplicates, by extracting liquid cultures 1:1 with ethyl acetate (EtOAc). MB cultures were grown at both stagnant and shaken conditions, whereas mannose and chitin were only grown at shaken conditions (200 rpm). The aqueous and organic phases were separated and the organic phase was evaporated under nitrogen. The dried extracts were re-dissolved in methanol (MeOH) prior to LC–MS analysis.

### Chemical analysis of secondary metabolites

Chemical analysis was performed using UHPLC-DAD-HRMS on a maXis 3G orthogonal acceleration quadrupole time-of-flight mass spectrometer (Bruker Daltonics) equipped with an electrospray ionization (ESI) source, as described by Holm et al*.*^66^. Data processing was performed using DataAnalysis 4.0 and Target Analysis 1.3 software (Bruker Daltonics).

### Fractionation of extracts for bioassays

Fractionation for bioassays was done on the combined extract from 8 × 1 l cultivations using chitin-based medium. The crude extract (520 mg) was fractionated by reversed phase (C18) chromatography on an Isolera One (Biotage) flash chromatography system, using a water-acetonitrile gradient running from 20–100% acetonitrile over 50 min with a flowrate of 15 ml/min using a 10 g column. Fractions were collected every 30 ml, and subsequently analyzed by UHPLC-DAD-HRMS and tested in inhibition assays (described below). Pure pseudane V and VII were obtained by subjecting the enriched fractions to semi-preparative HPLC on an Ultimate 3000 HPLC system (Dionex) equipped with a Kinetex C18, 2.6 μm, 2.1 × 100 mm column (Phenomenex) using a water-acetonitrile gradient of 10–100% acetonitrile over 15 min at a flowrate of 2 ml/min, at a temperature of 40 °C. Pure pseudanes were collected based on their absorption at 315 nm. Pure TBP was obtained as the retained fraction on an Isolute SCX (Waters) SPE column loaded with the TBP enriched fraction from the initial C18 fractionation. The column was equilibrated with 100% methanol followed by 70% methanol in water. The sample was loaded using 70% methanol and the column was washed with 70% methanol followed by 100% methanol. TBP was eluted using 2% ammonium hydroxide in methanol.

### Influence of substrate on pseudane and TBP production in strain S4498^T^

To determine how the carbon source affected the production of pseudanes and TBP in S4498^T^, the strain was grown in mannose, chitin, glucose, NAG and in MB media. Samples for CFU calculation were taken just before an aliquot of a 50 ml culture of each medium was diluted 1:1 in acetonitrile and centrifuged at 4200 RCF for 5 min on a Sigma 3–18 K centrifuge. Following centrifugation, each sample was analyzed by UHPLC-DAD-HRMS in negative ionization mode at the same conditions as described above. The relative amounts of pseudane V and TBP were determined using the integral of the extracted ion chromatograms for pseudane V ([M−H]^−^ = 214.1240) and TBP (TBP’s base peak of *m/z* = 381.6734 was used rather than [M−H]^−^ = 377.6767). The production of pseudane V and TBP was calculated as the area under the curve per CFU. A beeswarm boxplot was created in R (v3.4.2)^60^ and a Dunnett’s test was conducted to compare the production of pseudane V and TBP on the different media. The Dunnett’s test was performed with mannose as control.

### Testing for antibacterial activity

Target organisms for antibacterial susceptibility testing were *Vibrio anguillarum* 9011-286^67^ and Methicillin Resistant *Staphylococcus aureus* (MRSA) ATCC 33592. *V. anguillarum* and MRSA were grown aerated in 5 ml MB or Luria–Bertani broth (Difco, 244620) overnight and 60 µl culture was used to inoculate 60 ml medium containing 1% agar, 3% Instant Ocean Salts (Aquarium Systems), 0.3% casamino acids and 4% glucose. For MRSA, 1% peptone was added. Five mm wells were punched in the agar in which 60 µl extractions were added. Solvent was added as control. Plates incubated at 25 °C for 2 days and clearing zones were evaluated.

## Supplementary information

Supplementary Information.
