# Supplementary material for: Production of the antimicrobial compound tetrabromopyrrole and the Pseudomonas quinolone system precursor, 2-heptyl-4-quinolone, by a novel marine species Pseudoalteromonas galatheae sp. nov
Source: Sci Rep. 2020 Dec 10;10:21630. doi: 10.1038/s41598-020-78439-3 (PMC7730127; doi:10.1038/s41598-020-78439-3)

Supplementary information

**Production of the antimicrobial compound tetrabromopyrrole and the *Pseudomonas* quinolone system precursor, 2-heptyl-4-quinolone, by a novel marine species *Pseudoalteromonas galatheae* sp. nov.**

Sara Skøtt Paulsen, Thomas Isbrandt, Markus Kirkegaard, Yannick Buijs, Mikael Lenz Strube, Eva C. Sonnenschein, Thomas O. Larsen and Lone Gram*

**Figure S1.** Pigmentation of four type strains of yellow pigmented *Pseudoalteromonas flavipulchra* LMG20361, *P. piscicida* LMG 1142^T^, *P. peptidolytica* 14001^T^ and *P. maricaloris* LMG 19692^T^ and *P. galatheae* S4498^T^ (non-pigmented).

**Figure S2**. **Figure S2**. Phylogenetic tree based on 16S rRNA gene similarity from all *Pseudoalteromonas* type strains with validly published names in the genus. Species with a whole-genome sequenced representative are marked with an asterisk. Bootstrap values of >50 are included

**Figure S3**. Clustered heatmap based on glycosyl hydrolases (GHs) in the four yellow pigmented Pseudoalteromonas type strains and strain *P. galatheae* S4498^T^ and A757. Numbers in the matrix represent the number of GHs detected in the genomes.

**Figure S4.** Beeswarm boxplot of the production of pseudane V and tetrabromopyrrole (TBP) by *P. galatheae* S4498^T^ calculated as area under the curve per colony forming unit (AUC/CFU) when grown on mannose, chitin, marine broth (MB), glucose or NAG. A multiple comparison using the Dunnett’s test was performed using mannose as the control. All P-values less than 0.05 are summarized with an asterisk.

**Table S1.** Genomic comparison (percentage average nucleotide identity, ANI) of six strains of Pseudoalteromonas.

**Table S2.** In silico DNA-DNA hybridization values (DDH) for the type strains of *P. piscicida* NCIMB 1142^T^, *P. maricaloris* LMG 19692^T^ and *P. flavipulchra* LMG 20361^T^.

**Table S3.** Phenotypic characteristics of strain *P. galatheae* S4498^T^, *P. piscicida* NCIMB 1142^T^, *P. maricaloris* LMG 19692^T^, *P. flavipulchra* LMG 20361^T^ and *P. peptidolytica* DSM 14001^T^ as per a salt-modified BIOLOG Gen III MicroPlate™ assay.

[**Supplementary materials S1.** NMR tables and spectra for pseudane V and pseudane VII. 12](#_Toc42087161)

[**Supplementary materials S2**. a) Comparison of MS/MS fragmentation patterns and pathways for pseudane V and “pseudene V” at 20 and 40 eV CID (collision induced dissociation). b) Comparison of absorption spectra for pseudanes and pseudenes. 16](#_Toc42087162)

[**Supplementary materials S3.** Mass spectra showing the characteristic isotopic patterns for the halogenated pyrroles identified in *P. galatheae* S4498^T^. 17](#_Toc42087163)

[**Supplementary materials S4.** Base peak chromatograms of *P. galathea* S4498^T^, and each of the four type strains when grown on marine broth, with overlaid extracted ion chromatograms (EICs) of identified secondary metabolites. Secondary metabolites detected in trace amounts are not included as EICs. 18](#_Toc42087164)

[**Supplementary materials S5.** Mass spectra showing isotopic patterns or adduct patterns of identified secondary metabolites shown in Table 4, from the four type strains; *P. piscicida* NCIMB 1142^T^, *P. flavipulchra* LMG 20361^T^, *P. peptidolytica* DSM 14001^T^, and *P. maricaloris* LMG 19692^T^. 19](#_Toc42087165)

| **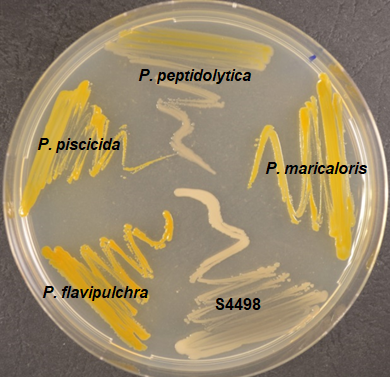** |
| --- |
| **Figure S1.** Pigmentation of type four strains of yellow pigmented Pseudoalteromonas flavipulchra LMG20361^T^, P. piscicida LMG 1142^T^, P. peptidolytica 14001^T^ and P. maricaloris LMG 19692^T^ and P. galatheae S4498^T^ (non-pigmented). |
|  |

**
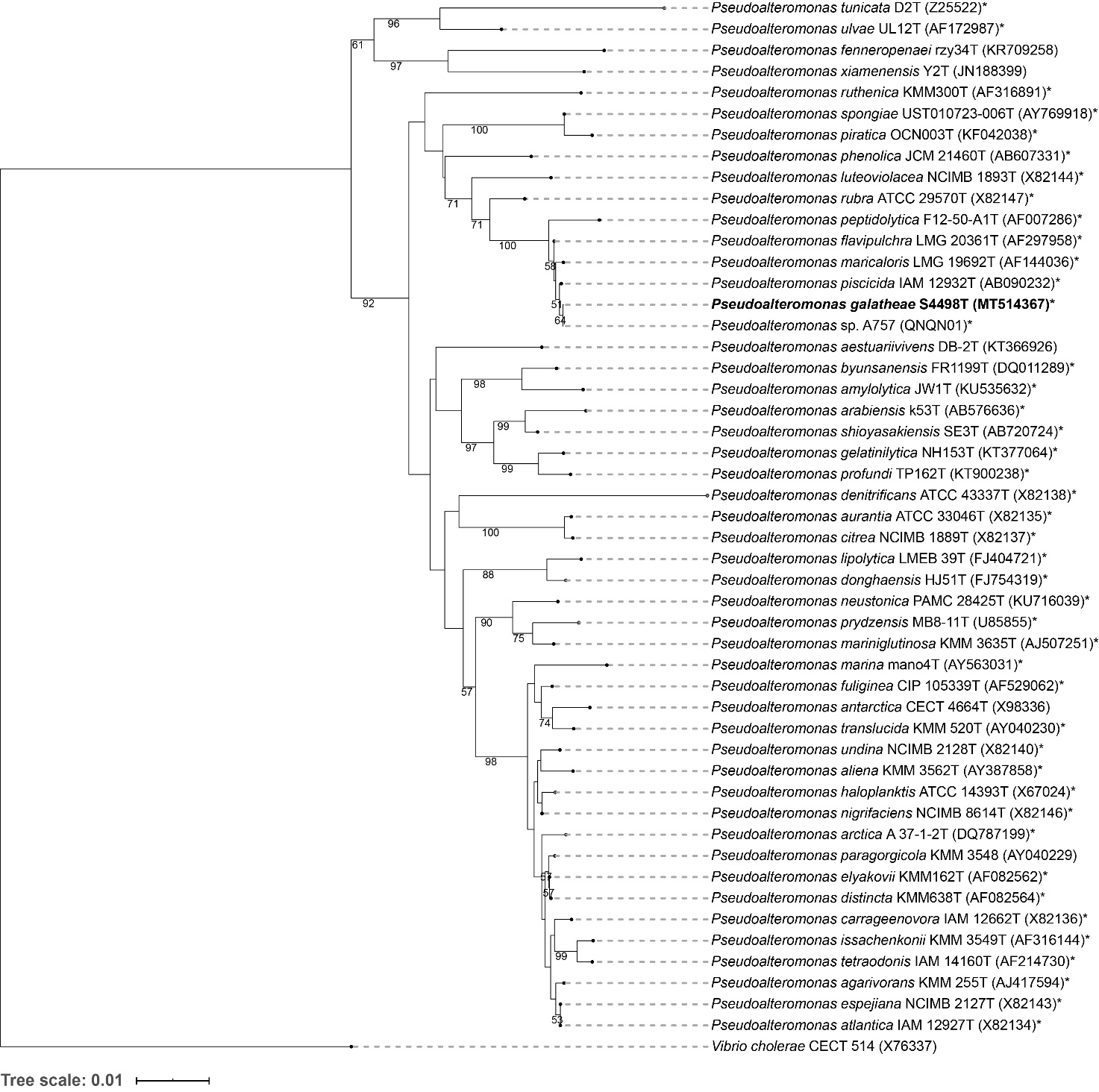
**

**Figure S2**. Phylogenetic tree based on 16S rRNA gene similarity from all *Pseudoalteromonas* type strains with validly published names in the genus. Species with a whole-genome sequenced representative are marked with an asterisk. Bootstrap values of >50 are included.


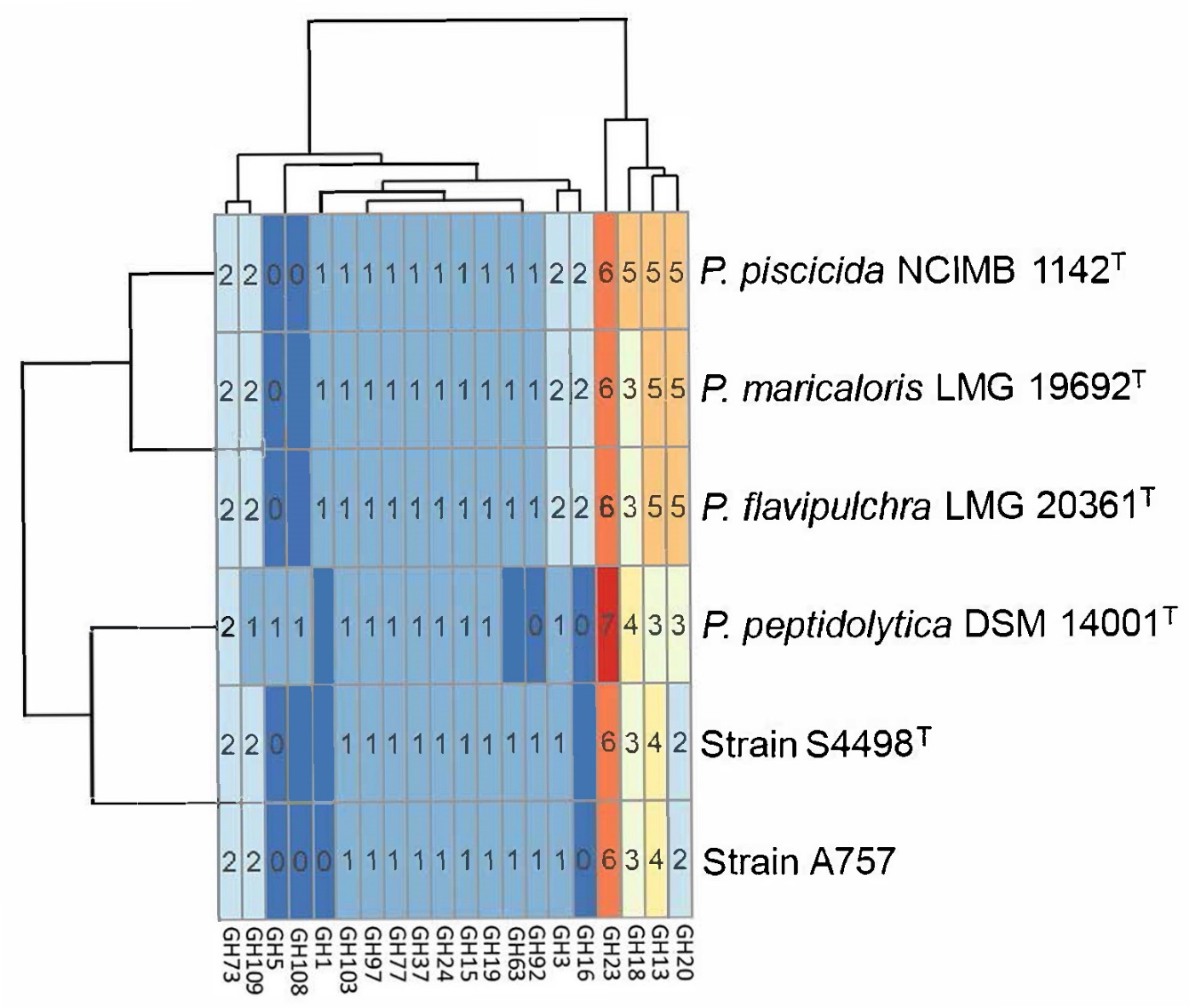


**Figure S3**. Clustered heatmap based on glycosyl hydrolases (GHs) in the four yellow pigmented Pseudoalteromonas type strains and strain P. galatheae S4498^T^ and A757. Numbers in the matrix represent the number of GHs detected in the genomes.

| 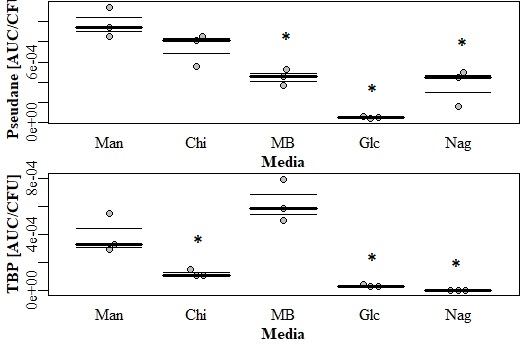 |
| --- |
| **Figure S4.** Beeswarm boxplot of the production of pseudane V and tetrabromopyrrole (TBP) by P. galatheae S4498^T^ calculated as area under the curve per colony forming unit (AUC/CFU) when grown on mannose, chitin, marine broth (MB), glucose or NAG. A multiple comparison using the Dunnett’s test was performed using mannose as the control. All P-values less than 0.05 are summarized with an asterisk. |

**Table S1.** Genomic comparison (percentage average nucleotide identity, ANI) of six strains of Pseudoalteromonas.

|  | Percentage average nucleotide identity, ANI | | | | | |
| --- | --- | --- | --- | --- | --- | --- |
|  | *P.peptidolytica* DSM 14001^T^ | *P.galatheae*  S4498^T^ | *P.maricaloris* LMG 19692^T^ | *P.piscicida* NCIMB 1142^T^ | *P.flavipulchra* LMG 20361^T^ | A757 |
| *P. peptidolytica* DSM 14001^T^ | 100 | 86.0 | 85.5 | 85.5 | 85.6 | 86 |
| *P. galatheae*  S4498^T^ | 86.0 | 100 | 93.1 | 93.0 | 93.2 | 99.3 |
| *P. maricaloris* LMG 19692^T^ | 85.5 | 93.2 | 100 | 96.4 | 98.8 | 93.1 |
| *P. piscicida* NCIMB 1142^T^ | 85.5 | 93.0 | 96.4 | 100 | 96.4 | 93.0 |
| *P. flavipulchra* LMG 20361^T^ | 85.6 | 93.2 | 98.8 | 96.4 | 100 | 93.2 |
| A757 | 85.6 | 99.3 | 93.1 | 93.0 | 93.2 | 100 |

**Table S2.** In silico DNA-DNA hybridization values (DDH) for the type strains of P. piscicida NCIMB 1142^T^, P. maricaloris LMG 19692^T^ and P. flavipulchra LMG 20361^T^.

| Strain comparison | DH estimate and range, % | Probability that DDH > 70% (i.e., same species) | Probability that DDH > 79% (i.e., same subspecies) |
| --- | --- | --- | --- |
| *P. piscicida* NCIMB 1142^T^ vs. *P. maricaloris* LMG 19692^T^ | 68.70% [65.7 - 71.6%] | 75.87% | 26.44% |
| *P. piscicida* NCIMB 1142^T^ vs. *P. flavipulchra* LMG 20361^T^ | 68.70% [65.7 - 71.5%] | 75.85% | 26.42% |
| *P. maricaloris* LMG 19692^T^ vs. *P. flavipulchra* LMG 20361^T^ | 89.60% [87.3 - 91.6%] | 95.67% | 64.69% |

**Table S3.** Phenotypic characteristics of strain P. galatheae S4498^T^, P. piscicida NCIMB 1142^T^, P. maricaloris LMG 19692^T^, P. flavipulchra LMG 20361^T^ and P. peptidolytica DSM 14001^T^ as per a salt-modified BIOLOG Gen III MicroPlate™ assay.

|  | ***P. galatheae* S4498^T^** | ***P. piscicida* NCIMB 1142^T^** | ***P. maricaloris* LMG 19692^T^** | ***P. flavipulchra* LMG 20361^T^** | ***P. peptidolytica* DSM 14001^T^** |
| --- | --- | --- | --- | --- | --- |
| Dextrin | + | + | + | + | + |
| D-Maltose | + | + | + | + | + |
| D-Trehalose | + | + | + | + | + |
| D-Cellobiose | + | + | - | - | + |
| Gentiobiose | + | + | + | + | + |
| Sucrose | + | + | + | + | + |
| D-Turanose | + | + | + | + | + |
| Stachyose | + | + | + | + | + |
| D-Raffinose | + | + | + | + | + |
| α-D-Lactose | + | + | + | + | + |
| D-Melibiose | + | + | + | + | + |
| β-Methyl-D-Glucoside | + | + | - | - | + |
| D-Salicin | + | + | - | - | + |
| N-Acetyl-D-Glucosamine | + | + | + | + | + |
| N-Acetyl-β-D-Mannosamine | + | + | + | + | + |
| N-Acetyl-D-Galactosamine | + | + | - | - | + |
| N-Acetyl Neuraminic Acid | + | + | + | + | + |
| α-D-Glucose | + | + | + | + | + |
| D-Mannose | + | + | + | + | + |
| D-Fructose | + | + | + | + | + |
| D-Galactose | + | + | + | + | + |
| 3-Methyl Glucose | + | + | + | + | + |
| D-Fucose | + | + | + | + | + |
| L-Fucose | + | - | + | + | + |
| L-Rhamnose | + | + | + | + | + |
| Inosine | + | + | + | + | + |
| D-Sorbitol | + | + | + | + | + |
| D-Mannitol | + | + | + | + | + |
| D-Arabitol | + | + | - | - | + |
| myo-Inositol | + | + | + | + | + |
| Glycerol | + | + | + | + | + |
| D-Glucose-6-PO4 | + | + | + | + | + |
| D-Fructose-6-PO4 | + | + | + | + | + |
| D-Aspartic Acid | - | + | - | - | - |
| D-Serine | - | + | - | - | - |
| Gelatin | + | - | + | + | + |
| Glycyl-L-Proline | + | + | + | + | + |
| L-Alanine | + | + | - | - | + |
| L-Arginine | + | - | + | + | + |
| L-Aspartic Acid | - | + | + | + | + |
| L-Glutamic Acid | - | + | + | + | + |
| L-Histidine | + | + | + | + | - |
| L-Pyroglutamic Acid | + | + | - | - | - |
| L-Serine | + | + | - | - | + |
| Pectin | + | + | + | + | + |
| D-Galacturonic Acid | + | + | + | + | - |
| L-Galactonic Acid Lactone | + | + | + | + | + |
| D-Gluconic Acid | + | - | + | + | - |
| D-Glucuronic Acid | + | + | + | + | - |
| Glucuronamide | + | + | + | + | + |
| Mucic Acid | + | + | + | + | + |
| Quinic Acid | + | + | + | + | + |
| D-Saccharic Acid | + | - | + | + | - |
| p-Hydroxy-Phenylacetic Acid | - | - | - | - | - |
| Methyl Pyruvate | - | - | - | - | - |
| D-Lactic Acid Methyl Ester | + | + | - | - | - |
| L-Lactic Acid | - | - | - | - | - |
| Citric Acid | + | - | + | + | - |
| α-Keto-Glutaric Acid | + | + | + | + | - |
| D-Malic Acid | - | - | - | - | - |
| L-Malic Acid | + | - | + | + | + |
| Bromo-Succinic Acid | - | + | - | - | - |
| Tween 40 | + | + | + | + | + |
| γ-Amino-Butryric Acid | - | + | - | - | - |
| α-Hydroxy-Butyric Acid | - | + | - | - | - |
| β-Hydroxy-D,L-Butyric Acid | + | + | - | - | - |
| α-Keto-Butyric Acid | + | + | + | + | + |
| Acetoacetic Acid | + | + | + | + | + |
| Propionic Acid | + | + | - | - | - |
| Acetic Acid | + | + | + | + | + |
| Formic Acid | - | + | - | - | - |
| pH 6 | + | - | + | + | + |
| pH 5 | - | - | - | - | - |
| 1% NaCl* | + | + | + | + | + |
| 4% NaCl* | + | + | + | + | + |
| 8% NaCl* | + | + | - | - | + |
| 1% Sodium Lactate | + | - | + | + | + |
| Fusidic Acid | + | - | + | + | + |
| D-Serine | + | - | + | + | - |
| Troleandomycin | - | - | - | - | + |
| Rifamycin SV | + | - | + | + | + |
| Minocycline | + | - | + | + | + |
| Lincomycin | + | + | + | + | + |
| Guanidine HCl | + | - | + | + | - |
| Niaproof 4 | - | - | - | - | - |
| Vancomycin | + | - | + | + | - |
| Tetrazolium Violet | - | - | - | - | - |
| Tetrazolium Blue | + | + | + | + | + |
| Nalidixic Acid | - | - | - | - | - |
| Lithium Chloride | + | + | + | + | - |
| Potassium Tellurite | + | - | + | + | - |
| Aztreonam | + | - | - | + | - |
| Sodium Butyrate | - | - | - | - | - |
| Sodium Bromate | - | - | - | - | - |

* The concentration is 2% higher, as the strains were inoculated with sea salt to accommodate growth

**Supplementary materials S1.** NMR tables and spectra for pseudane V and pseudane VII. NMR spectra were recorded on a Bruker Avance 800 MHz spectrometer (Bruker, Billerica, MA, USA). NMR spectra were acquired using standard pulse sequences. The solvent used was CD_3_OD, which was also used as reference with signals at δH = 3.31 ppm and δC = 49.0 ppm. Data processing and analysis was done using TopSpin 3.5pl7 (Bruker). J-couplings are reported in hertz (Hz) and chemical shifts in ppm (δ).

|  |  |  |  |  |  |  |
| --- | --- | --- | --- | --- | --- | --- |
| **Pseudane V** | |  |  | **Pseudane VII** | |  |
|  | ^1^H (δ, ppm) | ^13^C (δ, ppm) |  |  | ^1^H (δ, ppm) | ^13^C (δ, ppm) |
| 1 | 0.93 (t, J = 6Hz) | 14 |  | 1 | 0.9 (t, J = 6.9Hz) | 14 |
| 2 | 1.39 (m) | 23.1 |  | 2 | 1.31 (m) | 23.3 |
| 3 | 1.39 (m) | 32.2 |  | 3 | 1.31 (m) | 32.6 |
| 4 | 1.75 (m) | 29.6 |  | 4 | 1.38 (m) | 29.8 |
| 5 | 2.7 (t, J = 7.5Hz) | 34.7 |  | 5 | 1.42 (p , J = 7.5Hz) | 29.8 |
| 6 | - | 156.8 |  | 6 | 1.77 (dd , J = 7.7 / 7.7Hz) | 29.8 |
| 7 | 6.22 (s) | 108.5 |  | 7 | 2.72 ( , J = Hz) | 34.7 |
| 8 | - | 180.3 |  | 8 | - | n/a |
| 9 | - | 125.3 |  | 9 | 6.23 (s) | 108.5 |
| 10 | - | 141.4 |  | 10 | - | n/a |
| 11 | 7.57 (t, J = 8.3Hz) | 118.8 |  | 11 | - | n/a |
| 12 | 7.67 (t, J = 7.6Hz) | 133.1 |  | 12 | - | n/a |
| 13 | 7.38 (t, J = 7.6Hz) | 124.8 |  | 13 | 7.58 (dd, J = 8.3 / 1.4Hz) | 118.8 |
| 14 | 8.2 (dd, J = 8.1 / 1.2Hz | 125.6 |  | 14 | 7.69 (ddd , J = 8.3 / 7 / 1.4Hz) | 133.1 |
|  |  |  |  | 15 | 7.39 (ddd , J = 8.1 / 7, 1.2Hz) | 124.7 |
| 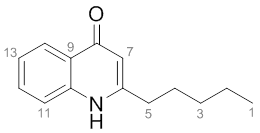 | | |  | 16 | 8.21 (dd , J = 8.1 / 1.2Hz) | 125.7 |
|  |  |  |  |  |  |  |
|  |  |  |  | 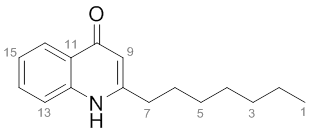 | |  |
|  |  |  |  |  |  |  |
|  |  |  |  |  |  |  |
|  |  |  |  |  |  |  |
|  |  |  |  |  |  |  |

**Pseudane V**


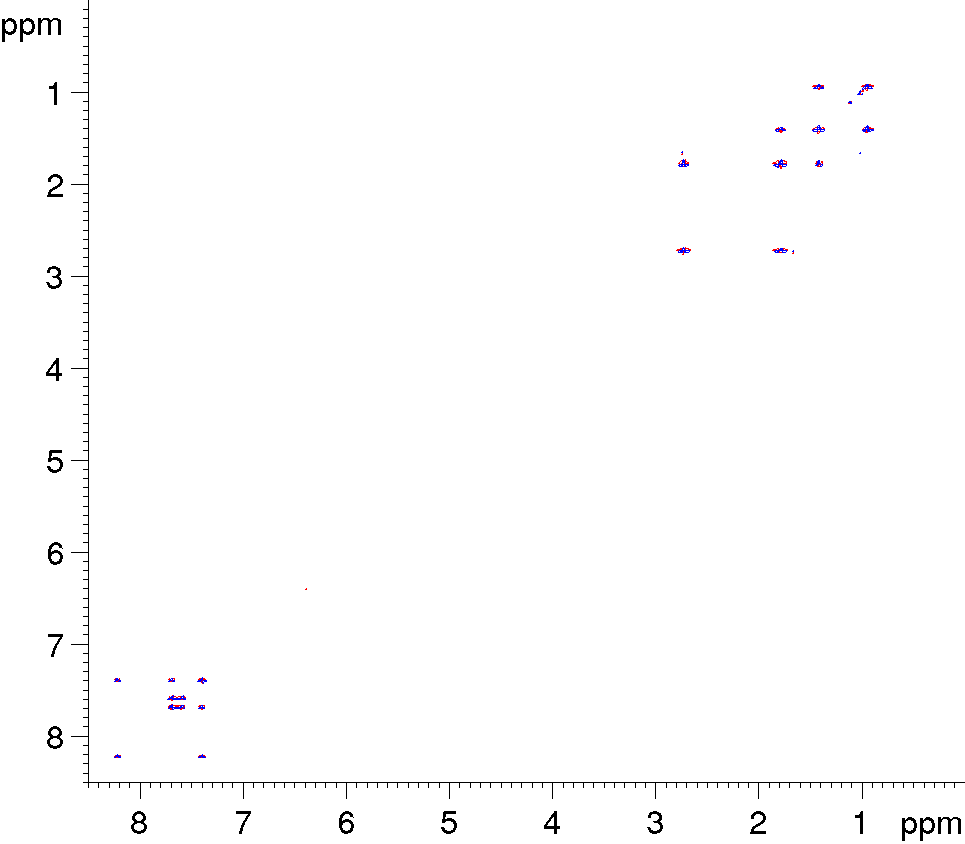


DQF-COSY


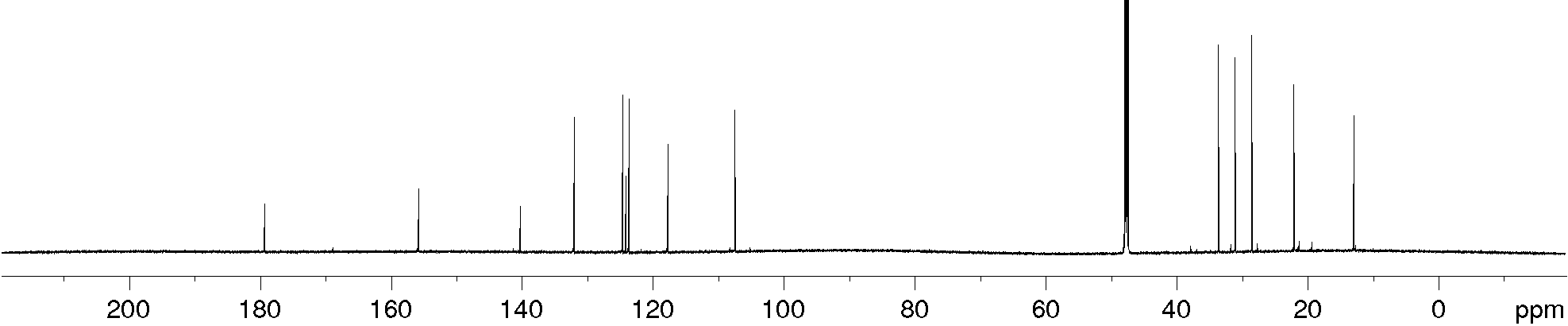


13C NMR


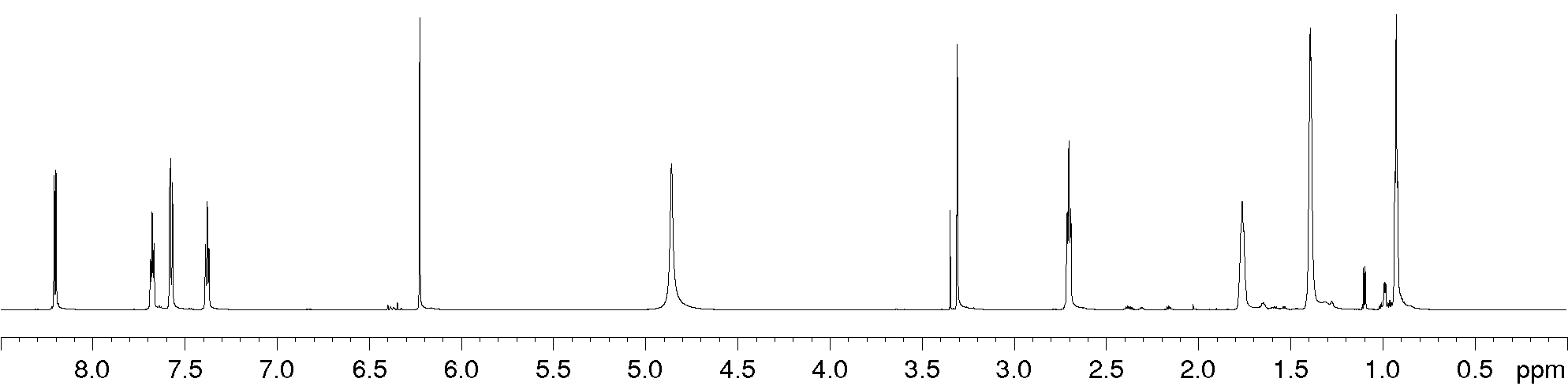


1H NMR

**Pseudane V**


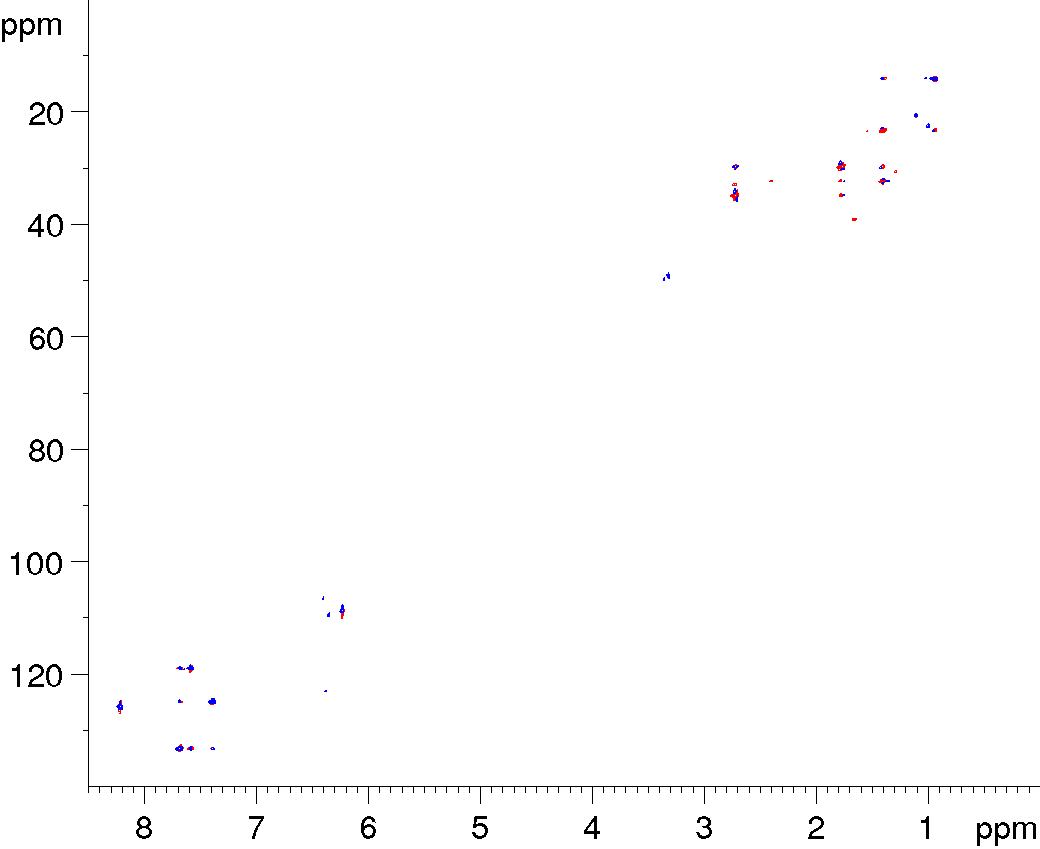


edHSQC


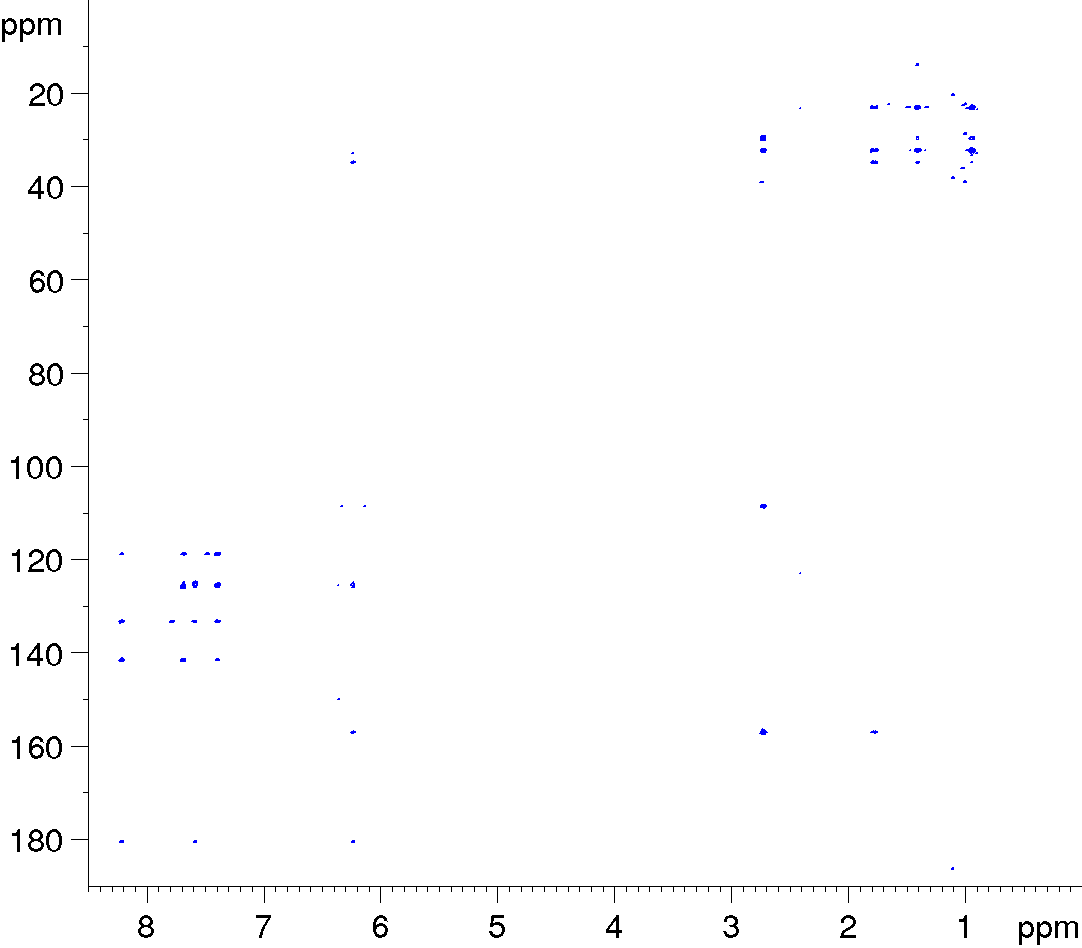


HMBC

**Pseudane VII**


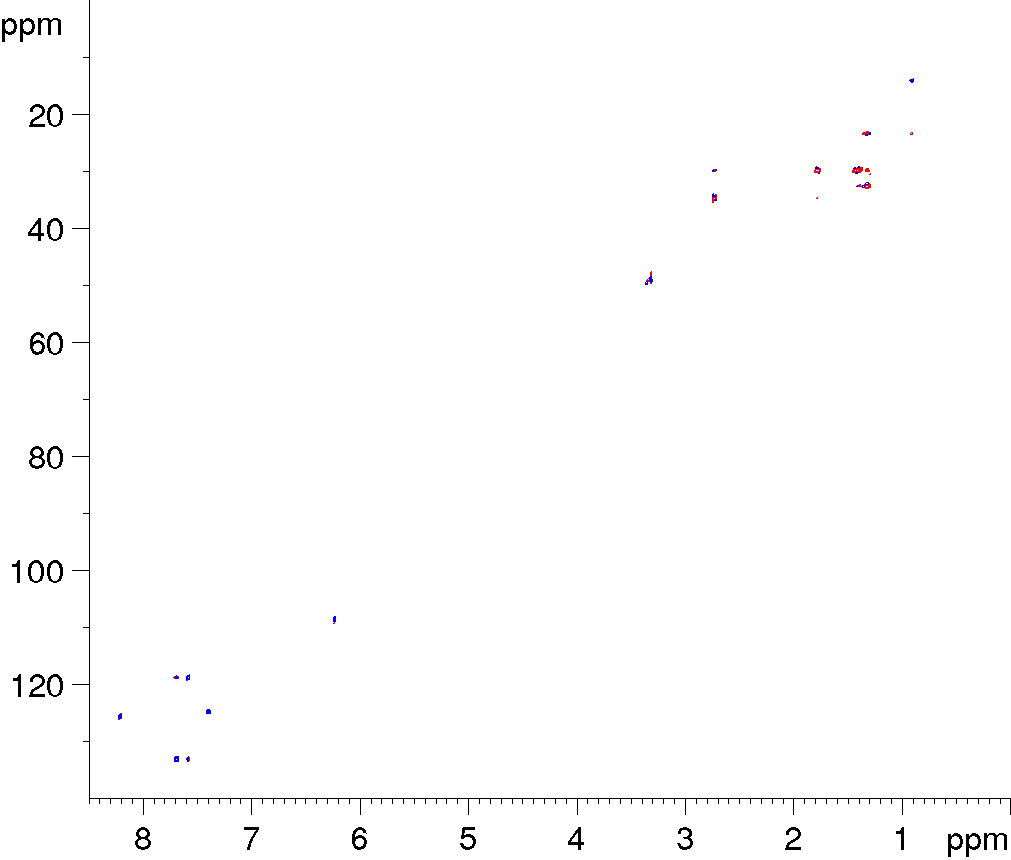


edHSQC


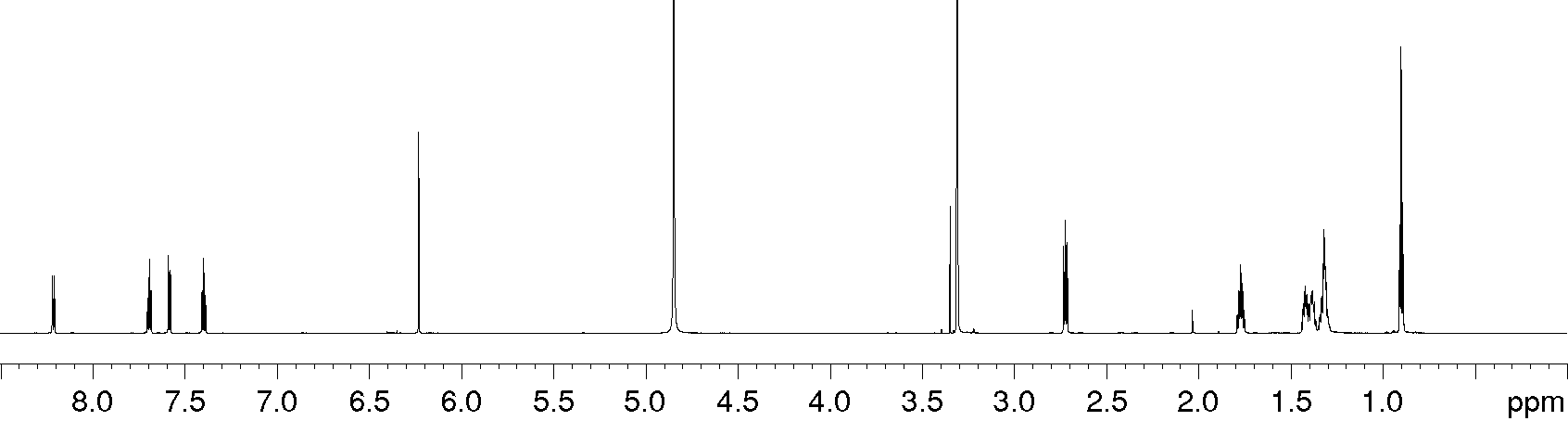


1H NMR


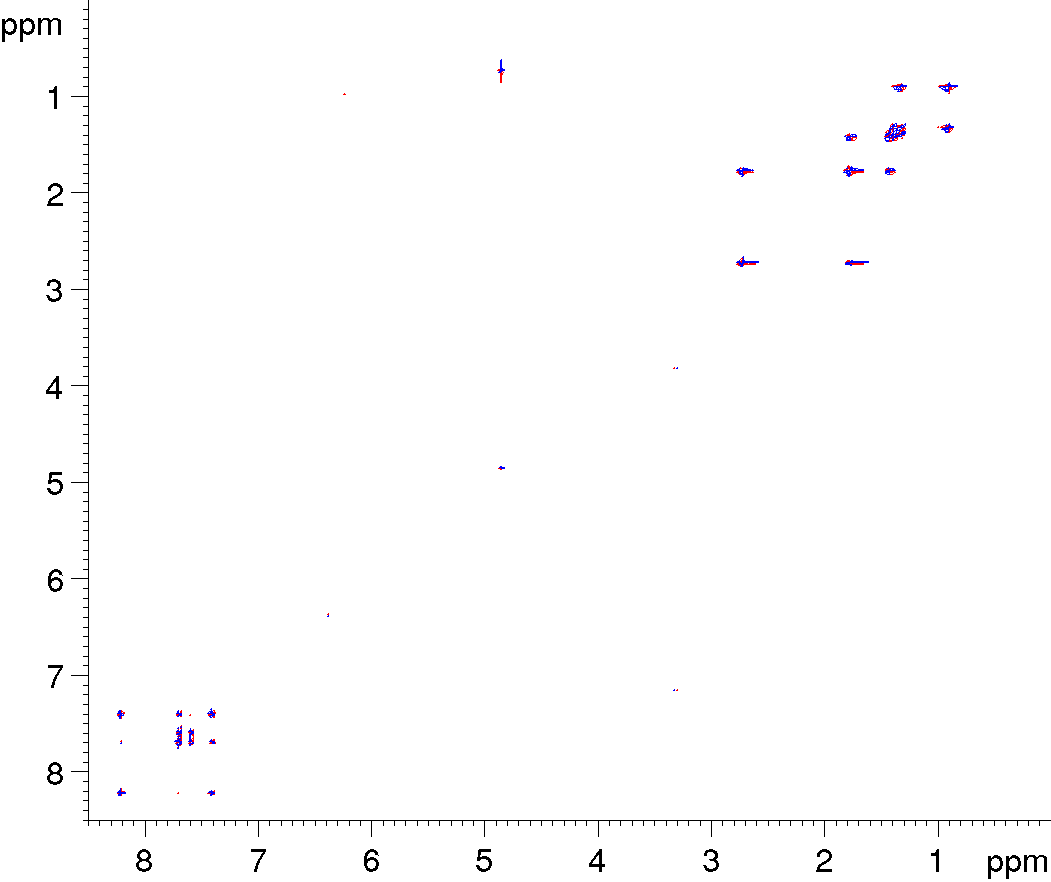


DQF-COSY

**Supplementary materials S2**. a) Comparison of MS/MS fragmentation patterns and pathways for pseudane V and “pseudene V” at 20 and 40 eV CID (collision induced dissociation). b) Comparison of absorption spectra for pseudanes and pseudenes.

**a)**


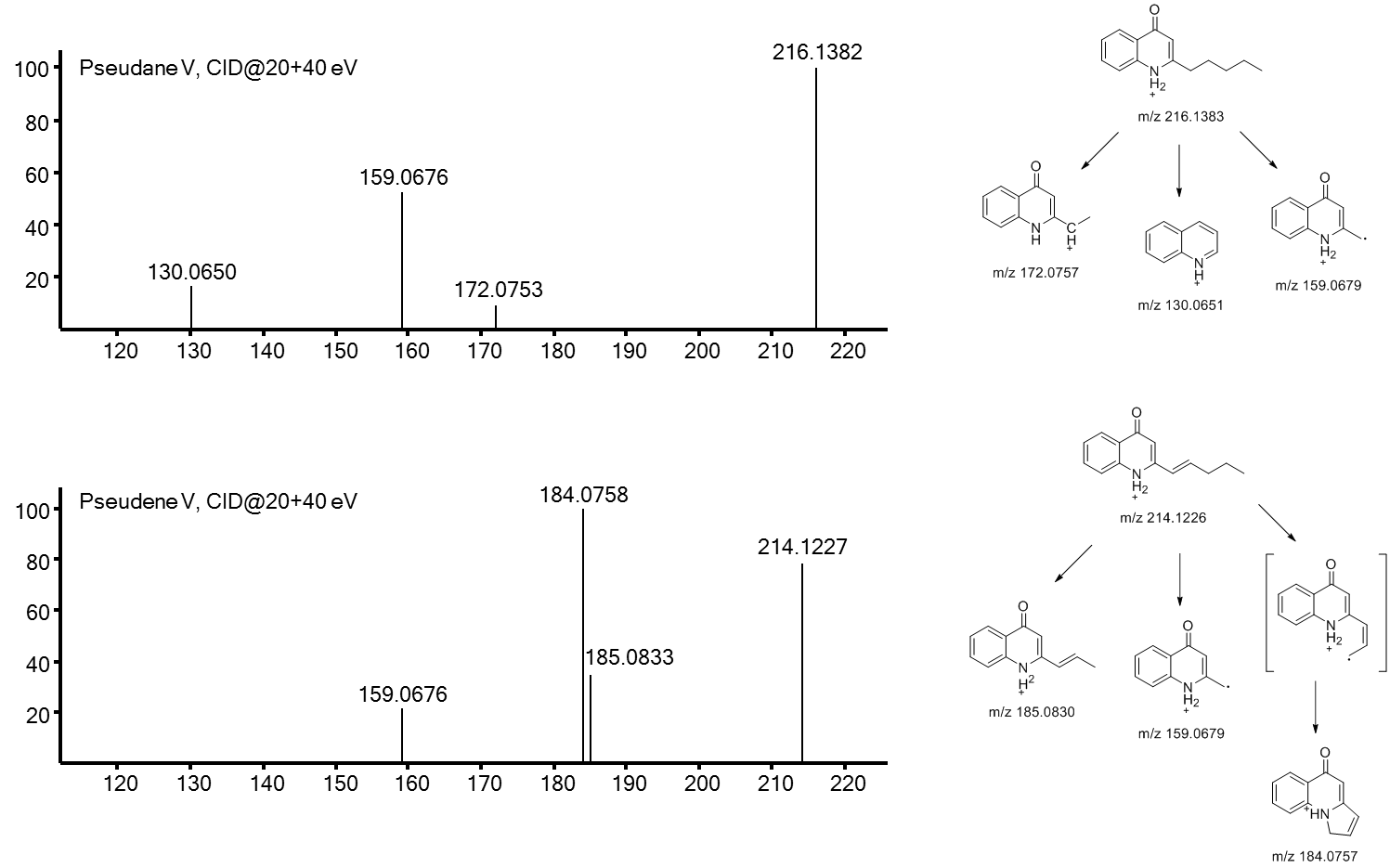


**b)**


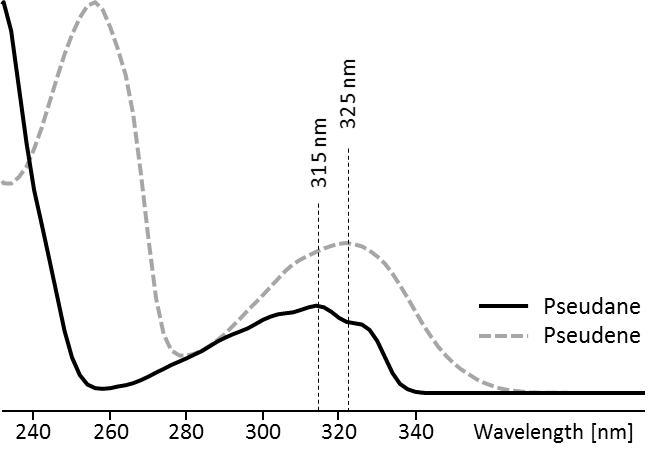


**Supplementary materials S3.** Mass spectra showing the characteristic isotopic patterns for the halogenated pyrroles identified in strain P. galatheae S4498^T^.


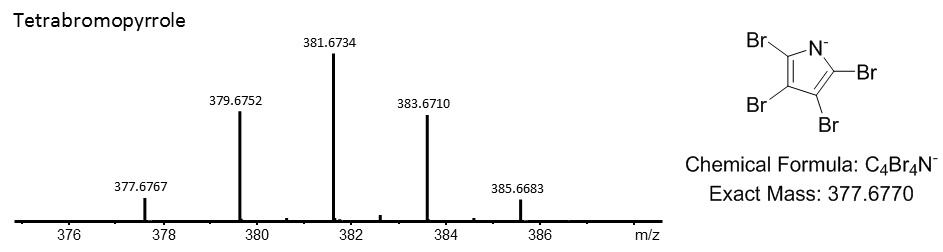


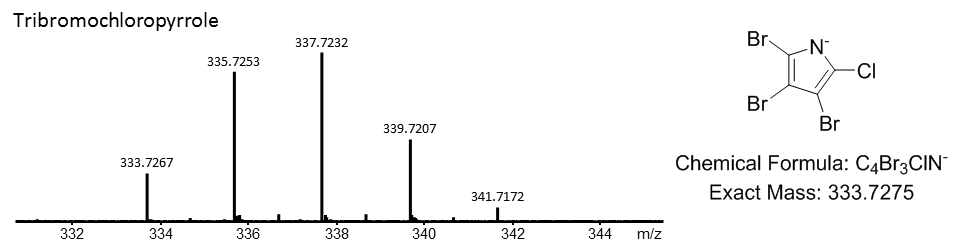


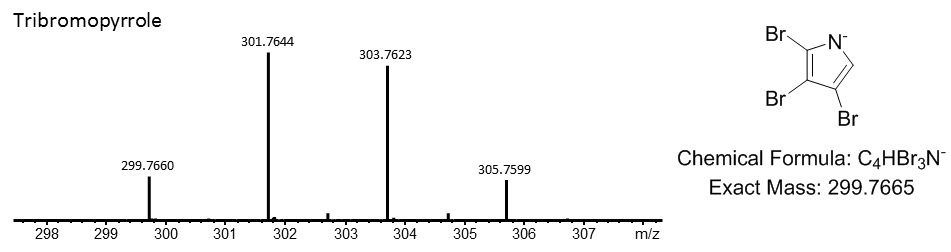


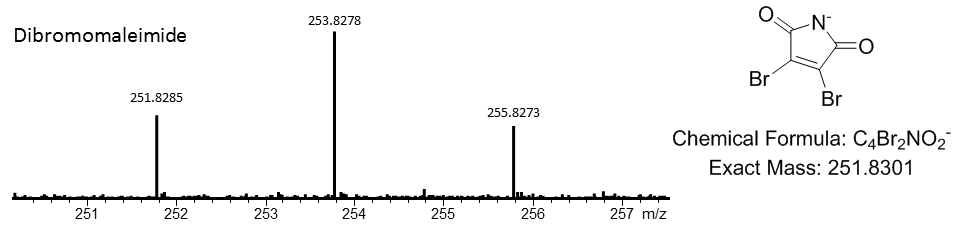


**Supplementary materials S4**
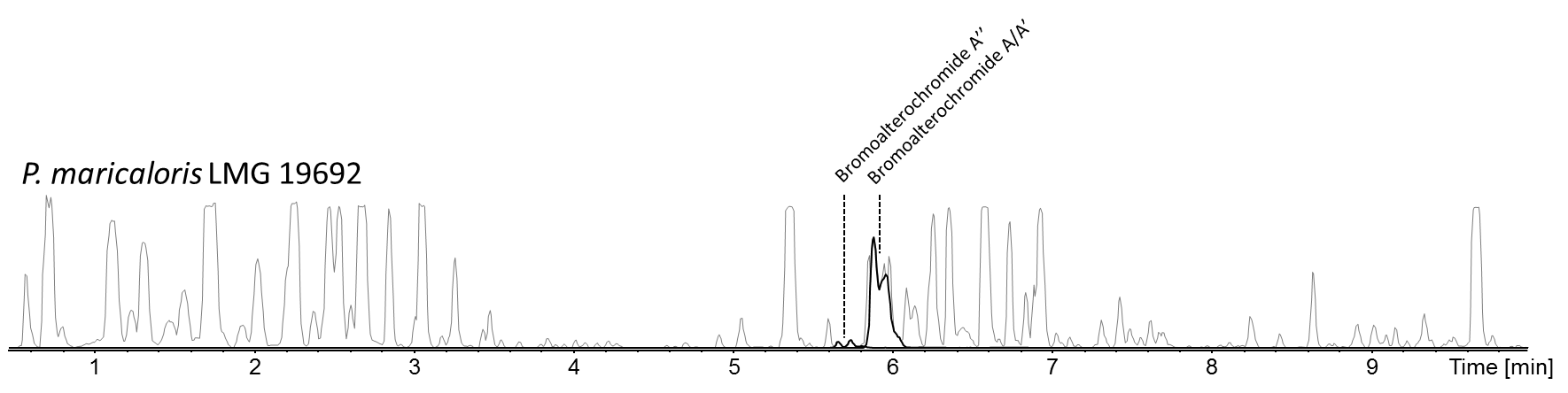

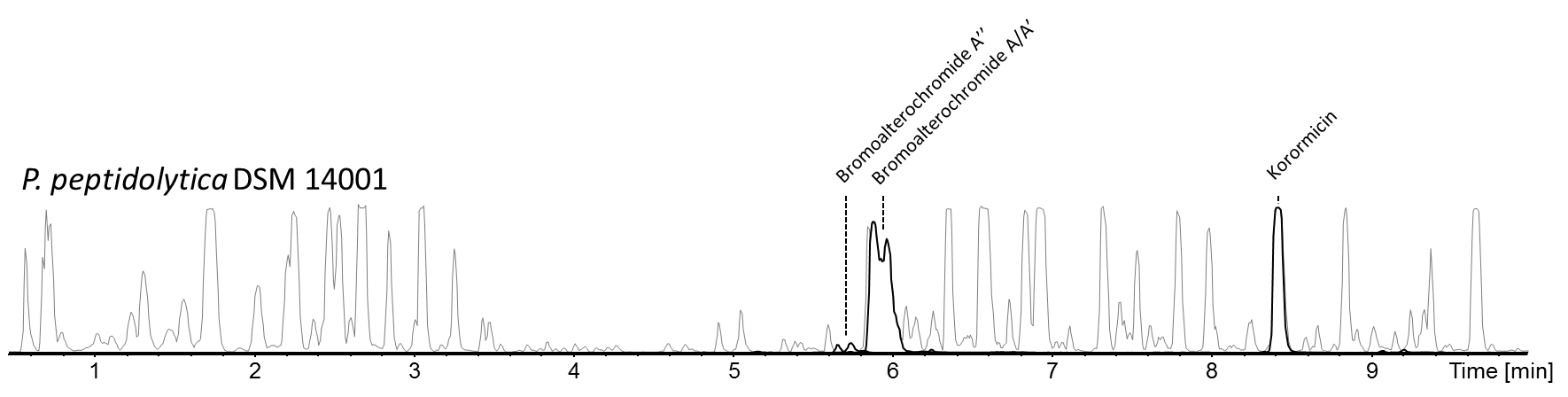

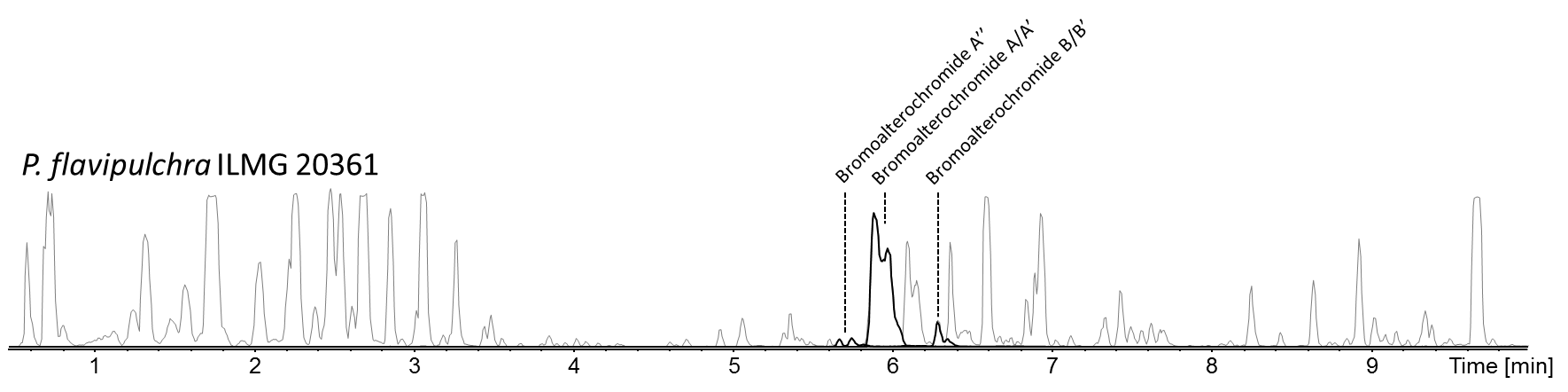

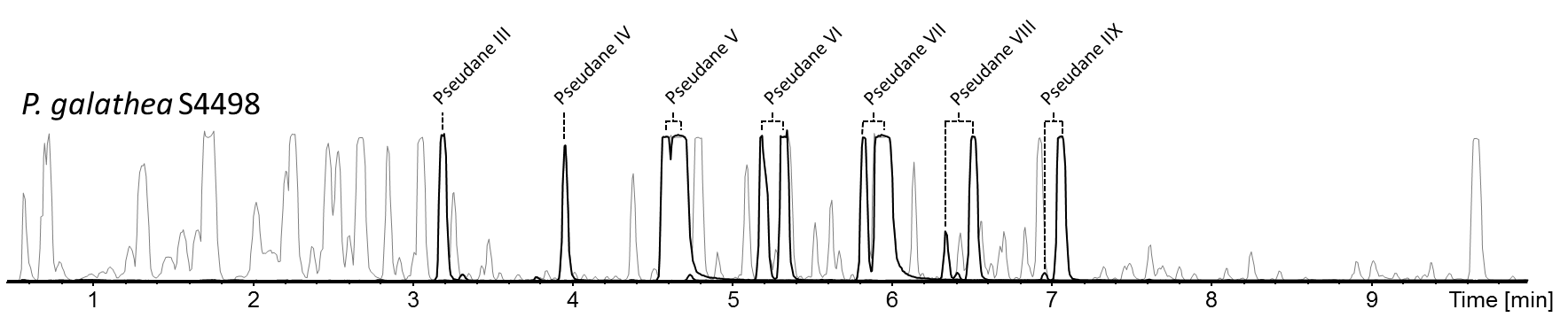

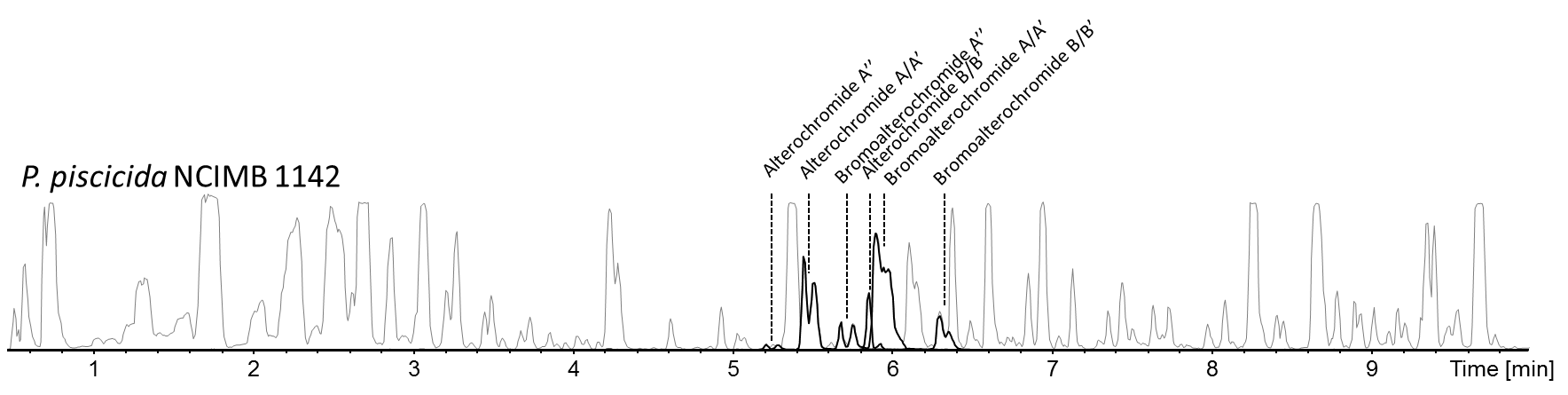
**.** Base peak chromatograms of P. galatheae S4498^T^, and each of the four type strains when grown on marine broth, with overlaid extracted ion chromatograms (EICs) of identified secondary metabolites. Secondary metabolites detected in trace amounts are not included as EICs.


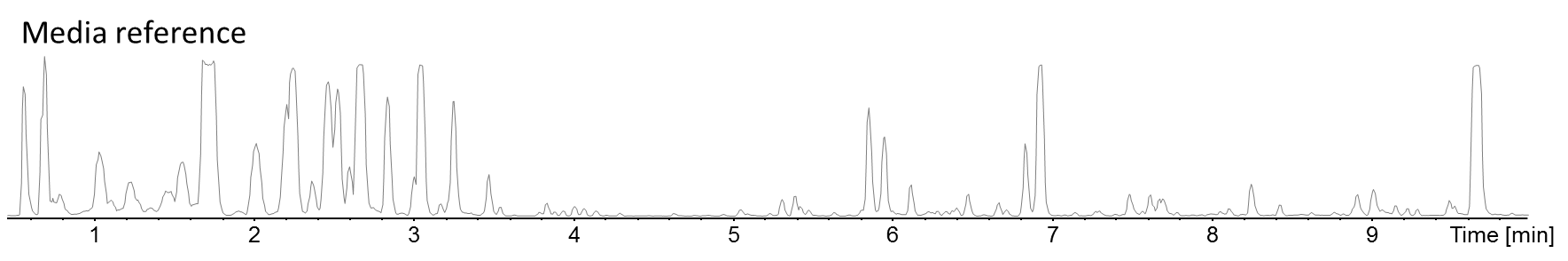


**Supplementary materials S5.** Mass spectra showing isotopic patterns or adduct patterns of identified secondary metabolites shown in Table 4, from the four type strains; P. piscicida NCIMB 1142^T^, P. flavipulchra LMG 20361^T^, P. peptidolytica DSM 14001^T^, and P. maricaloris LMG 19692^T^.


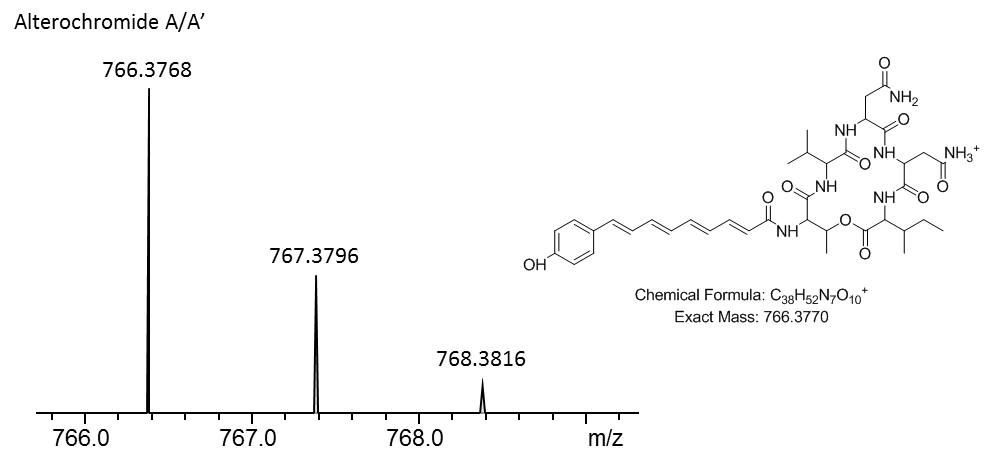


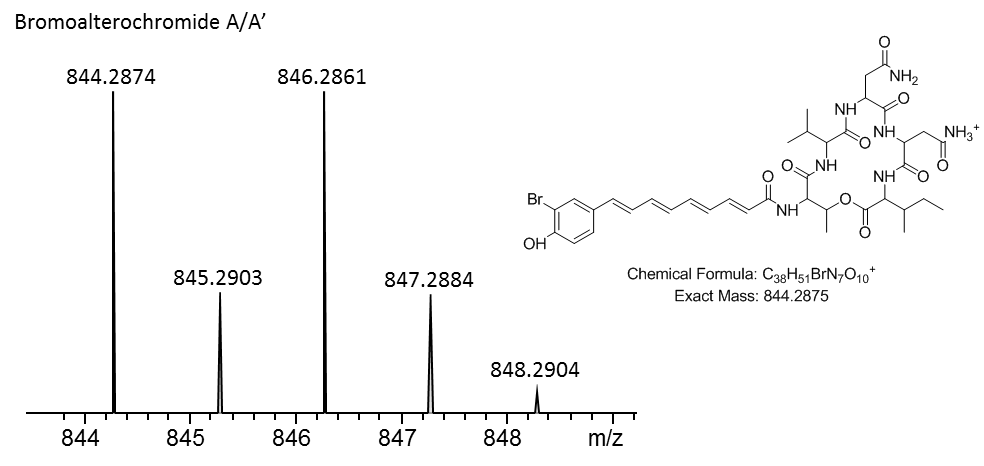


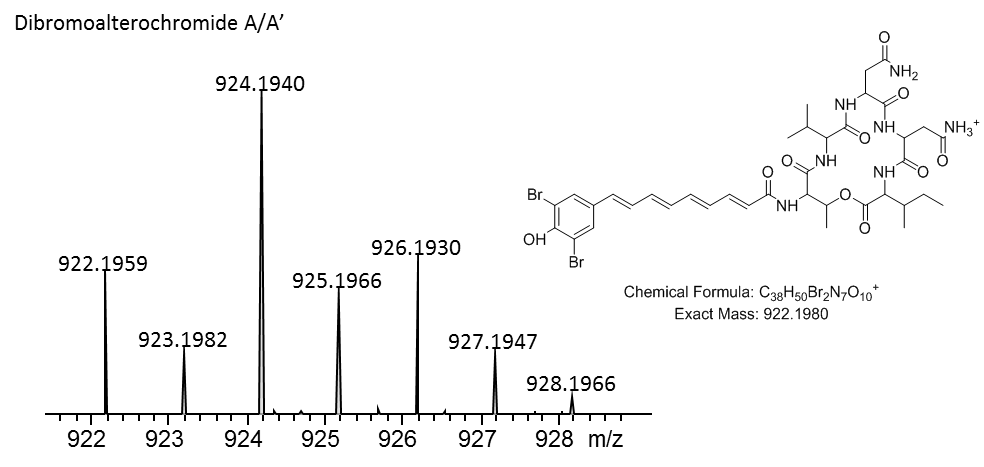


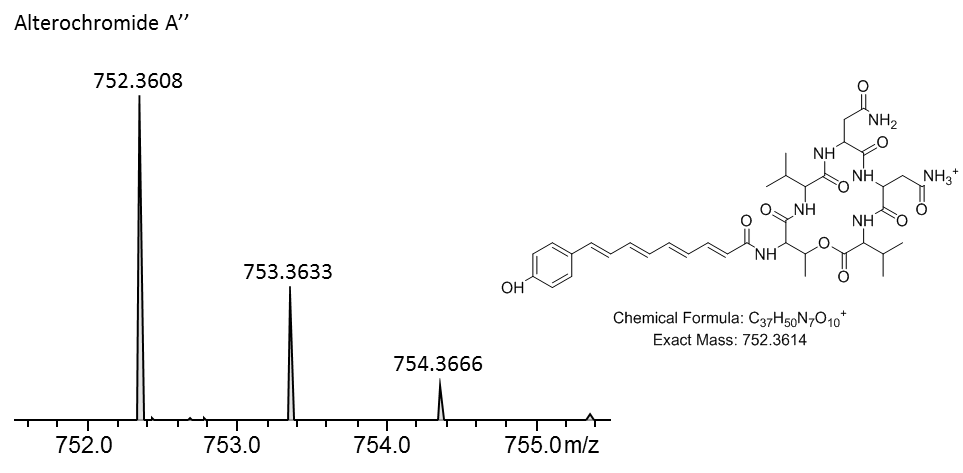


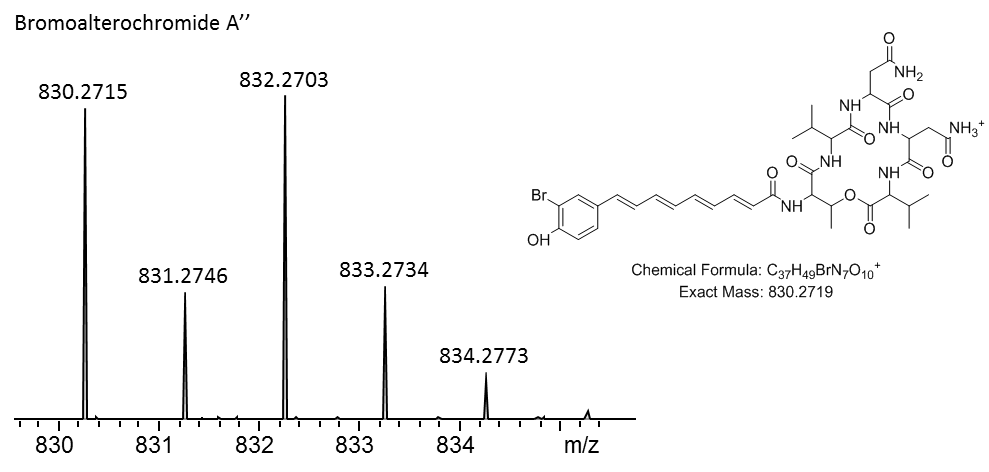


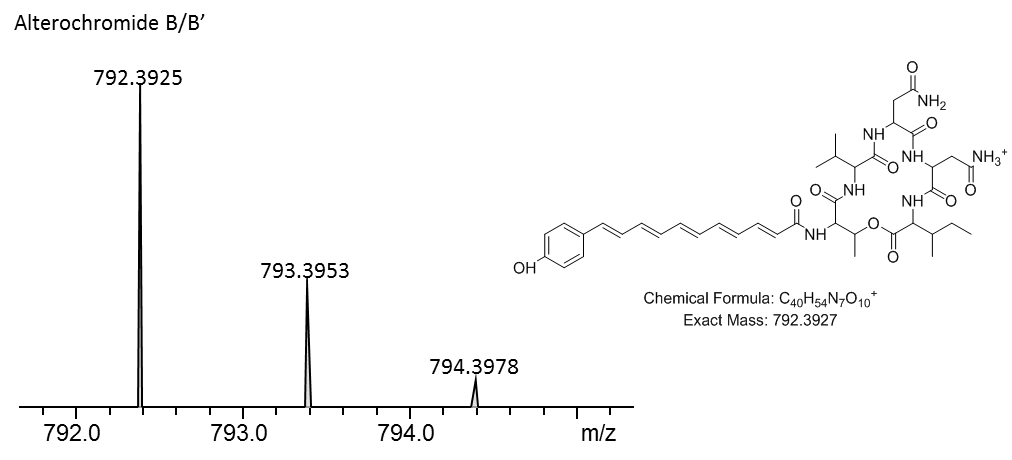


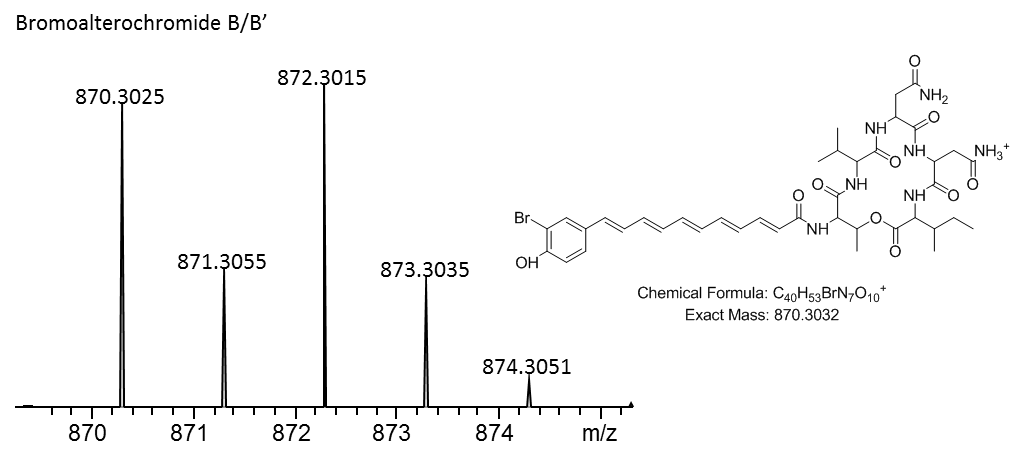


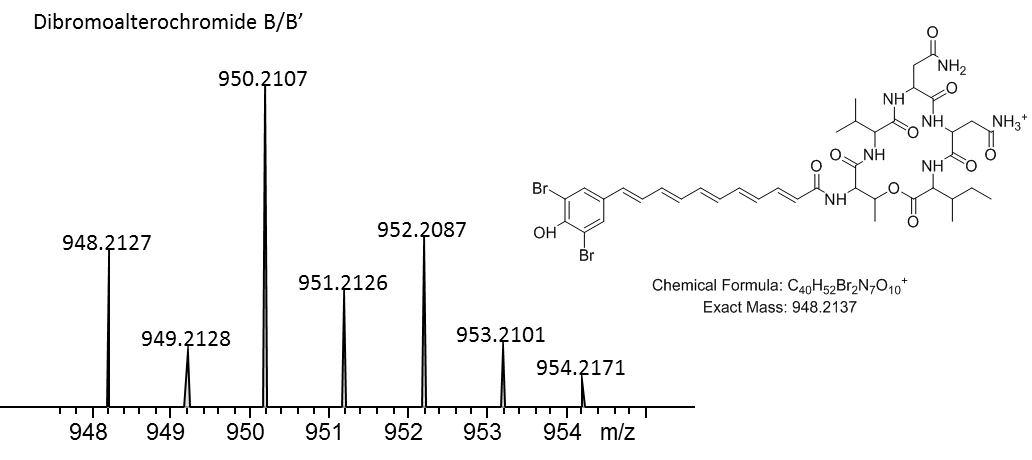


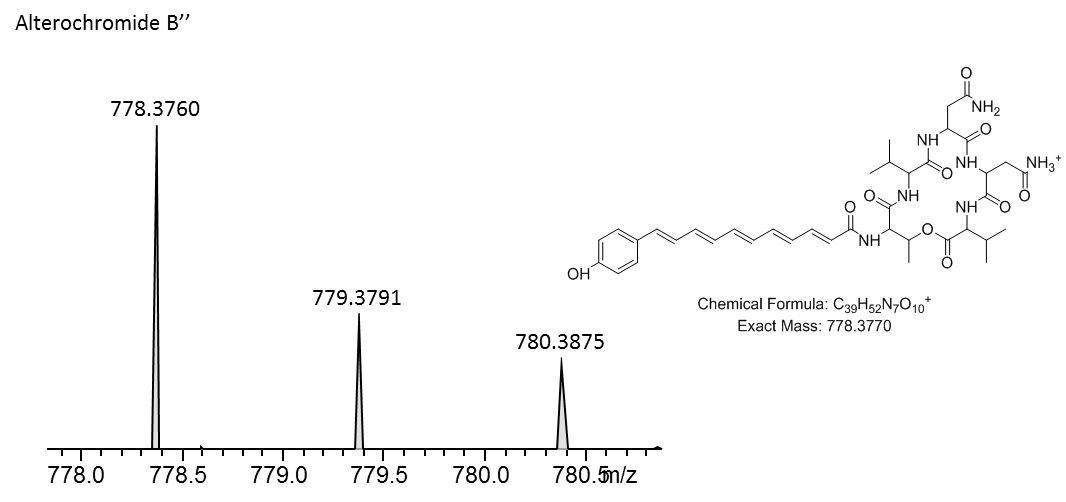


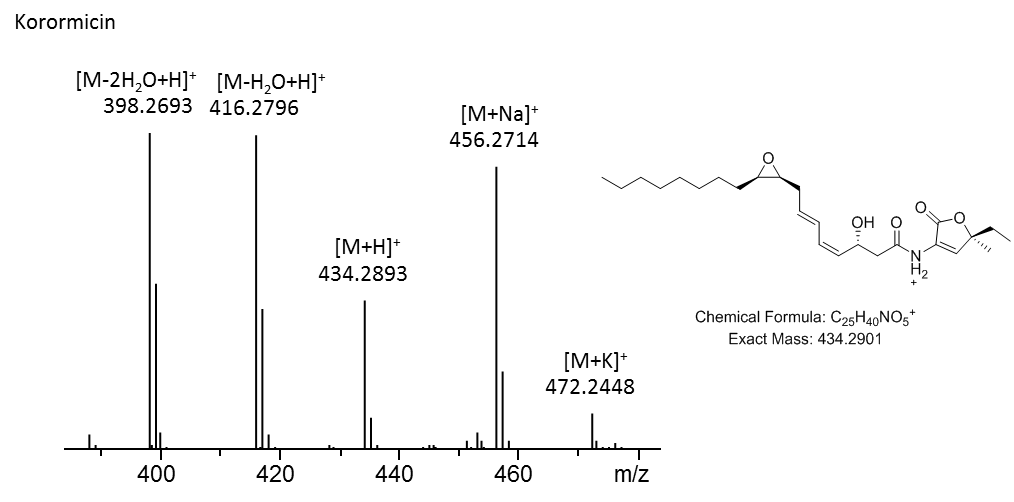

Supplement: Supplementary file 1 — Supplementary Information. [file 41598_2020_78439_MOESM1_ESM.docx]
